# Intrinsic PARG inhibitor sensitivity is mimicked by *TIMELESS* haploinsufficiency and rescued by nucleoside supplementation

**DOI:** 10.1093/narcan/zcae030

**Published:** 2024-07-16

**Authors:** Camilla Coulson-Gilmer, Samantha Littler, Bethany M Barnes, Rosie M Brady, Holda A Anagho, Nisha Pillay, Malini Dey, William Macmorland, Daniel Bronder, Louisa Nelson, Anthony Tighe, Wei-Hsiang Lin, Robert D Morgan, Richard D Unwin, Michael L Nielsen, Joanne C McGrail, Stephen S Taylor

**Affiliations:** Division of Cancer Sciences, School of Medical Sciences, Faculty of Biology, Medicine and Health, University of Manchester, Manchester Academic Health Science Centre (MAHSC), Manchester Cancer Research Centre, Wilmslow Road, Manchester M20 4GJ, UK; Division of Cancer Sciences, School of Medical Sciences, Faculty of Biology, Medicine and Health, University of Manchester, Manchester Academic Health Science Centre (MAHSC), Manchester Cancer Research Centre, Wilmslow Road, Manchester M20 4GJ, UK; Division of Cancer Sciences, School of Medical Sciences, Faculty of Biology, Medicine and Health, University of Manchester, Manchester Academic Health Science Centre (MAHSC), Manchester Cancer Research Centre, Wilmslow Road, Manchester M20 4GJ, UK; Division of Cancer Sciences, School of Medical Sciences, Faculty of Biology, Medicine and Health, University of Manchester, Manchester Academic Health Science Centre (MAHSC), Manchester Cancer Research Centre, Wilmslow Road, Manchester M20 4GJ, UK; Proteomics program, Novo Nordisk Foundation Center for Protein Research, Faculty of Health and Medical Sciences, University of Copenhagen, Copenhagen, Denmark; Division of Cancer Sciences, School of Medical Sciences, Faculty of Biology, Medicine and Health, University of Manchester, Manchester Academic Health Science Centre (MAHSC), Manchester Cancer Research Centre, Wilmslow Road, Manchester M20 4GJ, UK; Division of Cancer Sciences, School of Medical Sciences, Faculty of Biology, Medicine and Health, University of Manchester, Manchester Academic Health Science Centre (MAHSC), Manchester Cancer Research Centre, Wilmslow Road, Manchester M20 4GJ, UK; Division of Cancer Sciences, School of Medical Sciences, Faculty of Biology, Medicine and Health, University of Manchester, Manchester Academic Health Science Centre (MAHSC), Manchester Cancer Research Centre, Wilmslow Road, Manchester M20 4GJ, UK; Division of Cancer Sciences, School of Medical Sciences, Faculty of Biology, Medicine and Health, University of Manchester, Manchester Academic Health Science Centre (MAHSC), Manchester Cancer Research Centre, Wilmslow Road, Manchester M20 4GJ, UK; Division of Cancer Sciences, School of Medical Sciences, Faculty of Biology, Medicine and Health, University of Manchester, Manchester Academic Health Science Centre (MAHSC), Manchester Cancer Research Centre, Wilmslow Road, Manchester M20 4GJ, UK; Division of Cancer Sciences, School of Medical Sciences, Faculty of Biology, Medicine and Health, University of Manchester, Manchester Academic Health Science Centre (MAHSC), Manchester Cancer Research Centre, Wilmslow Road, Manchester M20 4GJ, UK; Genome Editing Unit, Faculty of Biology, Medicine and Health, University of Manchester, Michael Smith Building, Dover Street, Manchester M13 9PT, UK; Division of Cancer Sciences, School of Medical Sciences, Faculty of Biology, Medicine and Health, University of Manchester, Manchester Academic Health Science Centre (MAHSC), Manchester Cancer Research Centre, Wilmslow Road, Manchester M20 4GJ, UK; Department of Medical Oncology, The Christie NHS Foundation Trust, Wilmslow Rd, Manchester M20 4BX, UK; Division of Cancer Sciences, School of Medical Sciences, Faculty of Biology, Medicine and Health, University of Manchester, Manchester Academic Health Science Centre (MAHSC), Manchester Cancer Research Centre, Wilmslow Road, Manchester M20 4GJ, UK; Proteomics program, Novo Nordisk Foundation Center for Protein Research, Faculty of Health and Medical Sciences, University of Copenhagen, Copenhagen, Denmark; Division of Cancer Sciences, School of Medical Sciences, Faculty of Biology, Medicine and Health, University of Manchester, Manchester Academic Health Science Centre (MAHSC), Manchester Cancer Research Centre, Wilmslow Road, Manchester M20 4GJ, UK; Division of Cancer Sciences, School of Medical Sciences, Faculty of Biology, Medicine and Health, University of Manchester, Manchester Academic Health Science Centre (MAHSC), Manchester Cancer Research Centre, Wilmslow Road, Manchester M20 4GJ, UK

## Abstract

A subset of cancer cells are intrinsically sensitive to inhibitors targeting PARG, the poly(ADP-ribose) glycohydrolase that degrades PAR chains. Sensitivity is accompanied by persistent DNA replication stress, and can be induced by inhibition of *TIMELESS*, a replisome accelerator. However, the nature of the vulnerability responsible for intrinsic sensitivity remains undetermined. To understand PARG activity dependency, we analysed Timeless model systems and intrinsically sensitive ovarian cancer cells. We show that nucleoside supplementation rescues all phenotypes associated with PARG inhibitor sensitivity, including replisome speed and fork stalling, S-phase completion and mitotic entry, proliferation dynamics and clonogenic potential. Importantly nucleoside supplementation restores PARG inhibitor resistance despite the continued presence of PAR chains, indicating that sensitivity does not correlate with PAR levels. In addition, we show that inhibition of thymidylate synthase, an enzyme required for dNTP homeostasis, induces PARG-dependency. Together, these observations suggest that PARG inhibitor sensitivity reflects an inability to control replisome speed and/or maintain helicase-polymerase coupling in response to nucleotide imbalances.

## Graphical Abstract

**Graphical Abstract:**
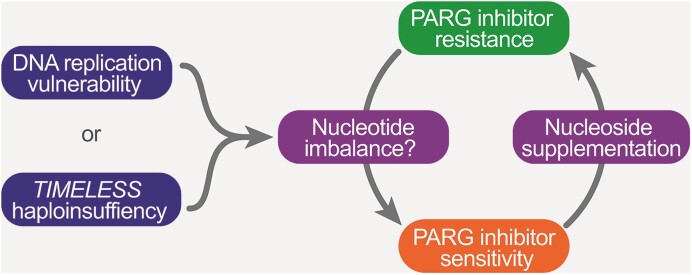

## Introduction

Targeting tumour-cell-specific cell cycle vulnerabilities represents a powerful anti-cancer strategy, highlighted by the spectacular success of poly(ADP-ribose) polymerase 1/2 inhibitors (PARPi) in the treatment of women with high-grade serous ovarian cancer (HGSOC), in particular HGSOC characterized by homologous recombination (HR) deficiency (1–4). However, at least 50% of ovarian cancers are HR-proficient and therefore less likely to respond to PARPi (5–7). Thus, there is a pressing need to identify drug targets that exploit vulnerabilities in HR-proficient ovarian cancer.

An emerging target is PARG, the poly(ADP-ribose) glycohydrolase that counterbalances PARP1/2-mediated poly(ADP-ribosyl)ation (PARylation) (8–14). A subset of ovarian cancer cell lines and patient-derived models are sensitive to PARG inhibitor (PARGi) monotherapy, and importantly sensitivity is neither mutually exclusive nor perfectly overlapping with PARPi-sensitivity, suggesting that a distinct cell cycle vulnerability leads to PARGi-sensitivity (12,15). Indeed, while PARGi-resistant cells tolerate poly(ADP-ribose) (PAR) chain stabilization, sensitivity is accompanied by persistent DNA replication stress leading to replication catastrophe, indicating an underlying DNA replication vulnerability, the nature of which remains undetermined. Interestingly, PARGi-mediated PAR chain stabilisation in *IDH1*-mutant glioma cells exposed to the alkylating agent temozolomide leads to metabolic catastrophe via sequestration of NAD^+^ (16). Whether metabolic and replication catastrophe phenotypes represent inter-related or distinct, context-dependent mechanisms is unknown. Therefore, dissecting the molecular mechanisms underpinning PARGi sensitivity is essential to drive development of predictive biomarkers needed to select patients for clinical trials.

To better understand the molecular determinants of PARGi sensitivity, we previously performed a *synthetic lethality* siRNA screen, identifying a number of DNA replication factors that, when inhibited, conferred PARGi sensitivity (12). One of the leading candidates, Timeless, along with Tipin and Claspin, is a component of the fork protection complex (FPC) (17). At active DNA replication forks, the FPC is positioned directly ahead of the replisome, physically engaging the CMG replicative helicase and DNA (18,19) where it functions to couple helicase-polymerase movement, maintain fork stability, facilitate CHK1-dependent signalling, and establish sister chromatid cohesion (20–32). In addition, Timeless is recruited to sites of DNA damage via interaction with PARP1 where it aids double-strand break repair (33,34). Timeless is also heavily PARylated in response to DNA damage (32,35).

Despite these insights, it remains unclear why repression of Timeless induces PARGi sensitivity. Moreover, whether synthetic lethality with Timeless reflects intrinsic PARGi-sensitivity is also unclear. Therefore, to further our understanding of the molecular mechanisms responsible for PARGi sensitivity, we took a two-pronged approach, analysing model systems engineered to be PARGi sensitive via inhibition of *TIMELESS*, while in parallel studying intrinsically sensitive ovarian cancer cells. We show that nucleoside supplementation rescues all of the molecular and cell cycle phenotypes associated with PARGi sensitivity, in both genetically engineered *TIMELESS* cells and intrinsically sensitive cells, but importantly without reverting PAR chain accumulation.

## Materials and methods

### Reagents, resources and contact for sharing requests

See Supplementary Table S1 for list of resources. Further information and reagent requests may be directed to Stephen S. Taylor (stephen.taylor@manchester.ac.uk).

### Experimental model and subject details

#### Patient sample collection

Research samples were obtained with informed patient consent from the Manchester Cancer Research Centre (MCRC) Biobank, which is licensed by the Human Tissue Authority (license number: 30004) and ethically approved as a research tissue bank by the South Manchester Research Ethics Committee (Ref: 22/NW/0237). MCRC Biobank's role is to distribute samples but does not endorse studies performed or the interpretation of results (see https://www.mcrc.manchester.ac.uk/research/mcrc-biobank). Ovarian cancer models (OCMs) 109 and 246 were previously established from platinum-resistant HGSOC samples, and detailed characteristics have been published (15,36).

### Human cell lines

RKO Flp-In™ T-Rex™ cells (37) and AAV293T cells (Agilent Technologies) cells were cultured in Dulbecco's modified Eagle's medium (DMEM). The human ovarian carcinoma cell lines OVCAR3 (ATCC), Kuramochi and OVMANA (JCRB Cell Bank) were cultured in RPMI 1640; COV362 and COV318 (Sigma-Aldrich) in DMEM. All were supplemented with 10% fetal bovine serum (FBS), 100 U/ml penicillin, 100 U/ml streptomycin and 2 mM glutamine and maintained at 37°C in a humidified 5% CO_2_ atmosphere. Established OCMs 109 and 246 were cultured in OCMI as described previously (38,39,40). FNE1 cells (kindly gifted by Dr Tan A. Ince) were cultured in WIT-Fo Culture Media (FOMI) at 5% O_2_ and 5% CO_2_ at 37°C (41). For long-term storage, cells were frozen in Bambanker (OCMs), 90% FBS 10% DMSO (cell lines), 20% FBS, 10% DMSO, 70% media (RKOs) and stored in LN_2_. Cells were periodically authenticated and tested for the presence of mycoplasma by the Molecular Biology Core Facility at the CRUK Manchester Institute.

### Materials and plasmids

PDD00017273 (PARGi) and Olaparib (PARPi) were dissolved in DMSO and used at a final concentration of 1 μM unless otherwise indicated. AZD6738 (ATRi), AZD7762 (Chk1i), AZD1775 (Wee1i), Camptothecin and EdU were dissolved in DMSO; Carboplatin, Hydroxyurea and Doxorubicin were dissolved in water; CldU and IdU were dissolved in culture media. Final agent concentrations are in figure legends. Thymidine and cytidine were made up in water, and adenosine and guanosine in DMSO. Due to cell line heterogeneity in terms of sensitivity to exogenous nucleosides, as also observed previously (42), we used 0.1 mM for RKO and 0.25 mM for the ovarian cancer cells. GST-tagged Af1521 macrodomain beads for ADPr-peptide enrichment were produced using BL21competent cells and coupled to glutathione Sepharose 4B beads (Sigma-Aldrich), as previously described (35,43,44).

### Generation of RKO cell lines expressing tetracycline-inducible *TIMELESS* transgenes

Synthetic *TIMELESS* cDNAs encoding wildtype (WT) and SH7A proteins, and resistant to multiple sgRNAs, were generated commercially (GeneArt, ThermoFisher Scientific). Site-directed mutagenesis was performed to (i) mutate serine 289 to alanine in WT, (ii) delete the 12 nucleotides encoding R^267^ALQ in WT and (iii) mutate the siRNA#1 and siRNA#3 binding sites in both WT and SH7A. cDNAs were cloned into pc5/FRT/TO/EmGFP and transformed into XL1-Blue competent cells. Plasmid DNA extracted using QIAprep Spin Miniprep Kit (Qiagen) was co-transfected with pOG44 (ThermoFisher Scientific) into RKO Flp-In^TM^ T-Rex^TM^ cells. Following selection in 400 μg/ml hygromycin B and 8 μg/ml blasticidin, colonies were pooled and expanded to create isogenic polyclonal cells.

### Generation of mutant *TIMELESS* cell lines using CRISPR/Cas9-mediated mutagenesis

RKO cells were seeded at 1.6 × 10^5^ cells/well in a 24-well plate for 24 h then transfected with a pD1301-based plasmid (Horizon Discovery), which expresses Cas9, a GPF-tag and a sgRNA targeting *TIMELESS*, using Lipofectamine 2000 according to manufacturer's instructions. After 48 h, cells were sorted by flow cytometry using a BD Aria II Cell Sorter (BD Biosciences) and GFP-positive cells seeded one cell/well in 96-well plates (Corning) to generate monoclonal cell lines.

To sequence *TIMELESS* transcripts RNA was extracted with the RNeasy Plus Mini Kit (Qiagen). cDNA spanning the sgRNA-targeting region was generated by RT-PCR using SuperScript™ III with Platinum™ Taq DNA polymerase (Qiagen) and a ProFlex PCR System (Thermo Fisher Scientific). PCR products were purified using the QIAquick PCR Purification Kit (Qiagen), digested overnight with *Sal*I and *Not*I and ligated into *Xho*I-*Not*I-digested pcDNA5/FRT/TO (ThermoFisher Scientific) and transformed into XL1 Blue *Escherichia coli* chemically competent cells.

### Targeting *TIMELESS* in FNE1 cells by CRISPR/Cas9-mediated mutagenesis

Using a Nucleofection SG Kit (Lonza), RNP complexes were formed by mixing: Buffer SG, a customized guide RNA targeting *TIMELESS* (sgTIMELESS_g015, design aided by WGE–CRISPR design tool, Wellcome Sanger Institute) and Cas9 (2:1, sgRNA:Cas9). 5 × 10^5^*TP53-*mutant FNE1 cells in RNP complex mix in a Nucleocuvette™ Vessel underwent pulse programme CM-137 of a 4D-Nucleofector® device (Lonza). RNP/cell mixture was plated with media in a 6-well plate (Corning), incubated at 5% O_2_ and 5% CO_2_ at 37°C and harvested 48 h post-nucleofection. After genomic DNA extraction using Purelink Genomic DNA mini kit (Thermo Fisher Scientific), the CRISPR-edited region was amplified using KOD Hot Start DNA Polymerase (Novagen) and a BioRad T100 Thermal Cycler. Purified PCR products underwent Sanger sequencing, and Insertion/Deletion (InDel) % analysed using Inference of CRISPR Edits (ICE) analysis tool (v3) (Synthego), which identified 43% of InDels. Following single-cell sub-cloning on the mixed pool and screening of clones, PT18 underwent amplicon-EZ-based next-generation sequencing (NGS).

pLVX-myc-GFP-H2B-Puro (12), underwent Gibson cloning to generate pLVX-myc-GFP-H2B-Neo. Wildtype or mutated (SH7A, S289A, Δ4) *TIMELESS* cDNA and pLVX backbone were digested using *Xho*I/*Not*I, and *TIMELESS* ligated in place of H2B. pLVX-myc-GFP-*TIMELESS*-Neo was transformed into XL1-Blue competent cells and plasmid DNA extracted using QIAprep Spin Miniprep Kit (Qiagen). Lentiviruses were produced by co-transfection of AAV293T cells at 1.2 × 10^6^ cells/well in a 6-well microplate, using a Promega ProFection Mammalian Transfection System kit. In each well: 1.5 μg Timeless construct, 2 μg psPAX2 and 0.5 μg pMD2.G (both kind gifts from Dr Didier Trono, EPFL, Lausanne, Switzerland). Transfection medium was replaced after overnight incubation and supernatant containing lentivirus harvested and pooled on the following two days, before being filtered (0.45 μm) and frozen at –80°C. Timeless construct-expressing cells (PT18+) were generated by transduction of PT18 with pLVX_Neo Timeless lentivirus (WT, Δ4, S289A or SH7A) in 12-well plates containing 200 or 400 μl lentivirus in media with 4 μg/ml polybrene, followed by centrifuging for 2.5 h at 30°C before 1 ml additional media added per well. Cells were subsequently grown in 0.8 mg/ml G418 to maintain a polyclonal cell population.

### Amplicon-EZ next-generation sequencing

For Amplicon-EZ NGS (Azenta Life Sciences) genomic DNA was extracted using PureLink™ Genomic DNA Mini Kit (Thermo Fisher Scientific) and nested PCR performed using RedTaq® DNA polymerase on a ProFlex PCR System (Thermo Fisher Scientific). For RKO Flp-In T-Rex *TIMELESS* cell lines, RNA was extracted using an RNeasy Plus Mini Kit (QIAGEN), and RT-PCR performed using Superscript III OneStep with Platinum Taq-HF and a ProFlex PCR System (Thermo Fisher Scientific). PCR fragments were purified using QIAquick PCR Purification Kit (QIAGEN). 500 ng in EB buffer was sent for library prep with Illumina® partial adapters and sequenced using an Illumina® NovaSeq X system (Illumina) at Azenta's European Genomics Headquarters in Leipzig, Germany. Further analysis was conducted using Integrative Genomics Viewer (IGV_2.16.1).

### Drug-sensitivity, proliferation and cell fate profiling using timelapse microscopy

Cells were seeded in black μclear® 96-well plates (Greiner Bio-One); cancer cell lines at 2000–5000 cells/well, FNE1s and FNE1-derived lines at 4500–10000 cells/well. Plates were imaged using an IncuCyte® Zoom or S3 (Sartorius AG) using 20x objective and maintained at 37°C in a humidified 5% CO_2_ (cell lines) or 5% CO_2_ and 5% O_2_ atmosphere (FNE1 and FNE1-derived cells). Phase contrast or fluorescence images were collected every 4 h (proliferation and drug sensitivity) or every 10 min (cell fate profiling). IncuCyte® ZOOM software and IncuCyte S3 cell-by-cell module were used in real time to measure green object count or for label-free counting, respectively. For cell fate profiling, image sequences were exported in MPEG-4 format and analysed manually to annotate cell behaviors and generate cell fate profiles (45). Data was imported into Prism 9 (GraphPad) for statistical analysis and presentation.

### Colony formation assay

Cells were seeded into 6-well plates at 500 cells/well and media containing drugs replaced every 3–4 days, with fixation in 2% (v/v) formaldehyde (Fisher Scientific). Cells were stained with 0.05% (w/v) crystal violet solution (Sigma-Aldrich), before imaging using a ChemiDoc™ Touch Imaging System (BioRad). Quantification of the colony area was performed using ImageJ software (NIH) with the ColonyArea plugin.

### Immunoblotting

Cell pellets were boiled in SDS buffer (0.35 M Tris pH 6.8, 0.1 g/ml sodium dodecyl sulphate, 93 mg/ml dithiothreitol, 30% (v/v) glycerol, 50 μg/ml bromophenol blue; all from Sigma Aldrich) and resolved by SDS-PAGE using NuPAGE™ 4–12% (v/v) Bis–Tris protein gels (1.0 mm) (Life Technologies), before electroblotting onto methanol-soaked Immobilon-P nitrocellulose membranes (Merck Millipore). Membranes were blocked in either 5% (w/v) dried skimmed milk (Marvel) or, for phospho-specific antibodies, 5% (w/v) bovine serum albumin (BSA, Sigma Aldrich) dissolved in TBST (50 mM Tris pH 7.6, 150 mM NaCl, 0.1% Tween-20). Membranes were incubated overnight at 4°C with primary antibodies (see Supplementary Table S1 – Bub3 1:1000; TIMELESS 1:1000; α-Tubulin 1:10 000; PCNA 1:1000; GFP-tag (D5.1) 1:1000), washed three times in TBS-T before incubating with the appropriate horseradish-peroxide (HRP)-conjugated secondary antibodies (Supplementary Table S1) for 2 hours. After three TBS-T washes, bound secondary antibodies were detected using either EZ-Chemiluminescence Reagent (Geneflow Ltd) or Luminata™ Forte Western HRP Substrate (Merck Millipore) and a ChemiDoc™ Touch Imaging System (BioRad). Image Lab software (BioRad) and Adobe Photoshop® CC 2018 (Adobe Systems Inc.) were used to process images.

For LI-COR immunoblotting, proteins were extracted, quantified by Bradford assay, and boiled in SDS buffer, resolved by SDS-PAGE and electroblotted onto Immobilon-FL PVDF membrane (Millipore; LI-COR) with REVERT total protein stain (LI-COR) used for loading normalization. Following imaging on the Odyssey® CLx Imaging System (Li-COR), REVERT was removed using reversal solution (0.1 M NaOH, 30% v/v methanol in water) and membrane blocked as above. Primary antibody incubation was performed as above (γ-tubulin 1:2000; Timeless 1:1000; Tipin 1:1000; pan-ADP-ribose 1:500; pKAP1 1:1000; γH2AX 1:1000). Membranes were then incubated with the appropriate fluorescently conjugated secondary antibodies (Supplementary Table S1) diluted 1:10 000 in 5% dried skimmed milk in 0.2% Tween-20, 0.01% SDS TBS, for 1 h. After rinsing with TBS, membranes were imaged on Odyssey® CLx Imaging System (LI-COR).

### Immunofluorescence

Cells were seeded onto 13 mm coverslips at 1.5–3 × 10^4^ cells/coverslip or 96-well Cell Carrier plates (PerkinElmer) at 2000–9000 cells/well. Cells were washed in PBS, fixed with 1% (v/v) formaldehyde for 5 min, washed in PBS, quenched with glycine for 5 min and washed in PBS-T (PBS, 0.1% (v/v) Triton X-100). For Timeless antibody, where stated, cells were pre-extracted for 2 min prior to fixation, by incubation on ice with ice-cold PBS containing 0.2% triton X100.

Cells were incubated with primary antibodies diluted in PBS-T (see Supplementary Table S1– γH2AX 1:2000; pKAP1 1:500; PAR (10H) 1:400; pan-ADPR 1:500; RPA70 1:500; Timeless 1:200) for 30 min at room temperature, washed and incubated with the appropriate fluorescently conjugated secondary antibodies (1:500) for 30 min at room temperature (see Supplementary Table S1), washed and DNA stained with 1 μg/ml Hoechst 33258 for 1 min at room temperature. Coverslips were mounted onto microscope slides (90% glycerol, 20 mM Tris, pH 9.2) and images acquired using either a 63× or 100× objective on an Axioskop2 (Zeiss, Inc) microscope fitted with a CoolSNAP HQ camera (Photometrics). MetaMorph Software (Molecular Devices) with Adobe Photoshop® CC 2018 (Adobe Systems Inc.) was used for image capture and processing. For high-throughput immunofluorescence, image acquisition used an Operetta® High Content Imaging System (PerkinElmer) with image analysis and quantitation using Harmony and Columbus High Content Imaging and Analysis Software (PerkinElmer). Mean fluorescence intensity or foci quantification within the nuclear area (demarcated using Hoechst stain) using ‘spot finder’ tool was quantified as a mean value per cell.

For EdU staining cells were labelled using the Click-iT Plus EdU Imaging kit (Invitrogen). In brief, cells were pulsed with 10 μM EdU in complete medium as specified in the figure legends then fixed with 3.7% (v/v) formaldehyde for 15 min, washed twice with 3% BSA in PBS and incubated with 0.5% Triton® X-100 in PBS for 20 min. Cells were then washed twice with 3% BSA in PBS, incubated with Click-iT® reaction cocktail for 30 min, and finally washed with 3% BSA in PBS. Primary and secondary antibody incubation and Hoechst staining was performed as described above.

### DNA fiber assays

Cells were incubated with 25 μM IdU for 20 min, followed by 250 μM CldU for 20 min, trypsinized and diluted in ice-cold PBS to 1.5 × 10^6^ cells/ml. 2 μl cell suspension was dropped onto microscope slides and dried at room temperature for 5 min before adding 7 μl spreading buffer (200 mM Tris–HCl pH 7.5, 50 mM EDTA, 0.5% SDS). After 5 min slides were tilted approximately 10–20° and spread fibers air dried and fixed in methanol/acetic acid (3:10) for 10 min, dried and stored at 4°C. Fibers were denatured in 0.5 M NaOH, 1 M NaCl for 8 min at room temperature and washed three times in PBS, dehydrated in 70%, 90% and 100% EtOH in succession for 3 min each, followed by drying at room temperature. For immunolabelling, all antibodies were dissolved in BlockAid^TM^ Blocking Solution (Thermo Scientific) and slides incubated with a rat anti-BrdU antibody (Abcam) to detect CldU alongside a mouse anti-BrdU antibody (BD Biosciences) to detect IdU (see Supplementary Table S1) for 1 h at 37°C and then washed three times with 1x PBS-0.05% Tween-20. Slides were then incubated with goat anti-rat Cy5 and goat anti-mouse Cy3.5 antibodies (both Abcam, Supplementary Table S1) for 45 min at 37°C, followed by three washes with 1x PBS-0.05% Tween-20 and three washes with PBS. Slides were dehydrated with 70%, 90%, 100% EtOH in succession for 3 min each, then mounted onto coverslips using ProLong Gold antifade reagent (Thermo Scientific). Images were acquired using Axioskop2 (Zeiss, Inc.) microscope fitted with a CoolSNAP HQ camera (Photometrics) and 2–5 slides analysed per condition. Fiber lengths were quantified using ImageJ software (NIH) and used to evaluate replication fork speed (Note, 1 μm corresponds to 2.59 kb) (46,47).

### TIPIN antibody production

A cDNA fragment encoding human TIPIN was amplified using Platinum™ *Taq* DNA polymerase (Invitrogen) and ligated into a pGex-4T-3 vector (GE Healthcare). The GST-TIPIN fusion protein, expressed in *E. coli* BL21 competent cells using 1 mM IPTG whilst shaking at 37°C for 2 h, was purified using glutathione sepharose beads (Expedeon) and used for sheep immunisation (Alta Bioscience). Polyclonal sheep anti-TIPIN antibodies were purified from serum by affinity purification according to standard procedures. In brief, serum was first passed through covalently coupled GST-glutathione sepharose beads to remove anti-GST antibodies. Covalently coupled GST-TIPIN beads were incubated with the serum, bound anti-TIPIN antibodies were eluted with 100 mM glycine (pH 2.8), neutralised using 1 M sodium phosphate (pH 8.0), and dialysed against PBS.

### RNA interference

Reverse transfection used Opti-MEM media, DharmaFECT-1 transfection reagent (Dharmacon/Horizon Discovery) and siRNA at a final concentration of 66 nM, according to manufacturer's instructions (all SMARTpool consisting of four individual ONTARGETplus oligonucleotides from Horizon Discovery, as described in Supplementary Table S2). Cells were mixed with transfection reagents and seeded into 96-well Cell Carrier plates (PerkinElmer) at the following densities/well: RKO, 8000; COV318, 5500; COV362, 4000; OVCAR3, 3500. Time courses and drug additions are stated in figure legends. Cells were fixed and stained according to the immunofluorescence protocol using an Operetta® High Content Imaging System (PerkinElmer).

For the RNAi complementation assay, RKO Flp-In™ T-Rex™ *TIMELESS* WT and mutant cells underwent reverse transfection as above, with cells seeded at 2000 cells/well in a 96-well Cell Carrier plates (PerkinElmer) incubated for 24 h. Cells were re-transfected ± 500 ng/ml tetracycline, incubated for 24 h, before a further 48 h incubation with 1 μM PARGi ± 500 ng/ml tetracycline and immunofluorescence performed as above. For immunoblotting cells were transfected and seeded as above, incubated for 48 h, treated ± 500 ng/ml tetracycline and incubated for a further 24 h before harvesting as described in the immunoblotting protocol.

### iPOND

The protocol followed that described previously with minor modifications (48). Cells were seeded in 15 cm dishes and treated with 1 μM PARGi for 24 h to give 40 × 10^7^ cells/condition when cells were pulsed with 10 μM EdU for 20 min. Cells were crosslinked with 1% formaldehyde for 10 min, quenched with 1.25 M glycine, washed three times with PBS, permeabilized with 0.25% Triton X-100 in PBS for 30 min then washed three times with PBS. Cells were incubated with click reaction buffer for 2 h at room temperature (20 μM biotin azide, 10 mM sodium ascorbate and 2 mM CuSO_4_ in PBS), followed by two washes with PBS and resuspension in lysis buffer (50 mM Tris–HCl, pH 8, 1% SDS). Chromatin was sonicated at 4 °C using a microtip QSonica Q125 Sonicator for 20 s constant pulse at 30% amplitude followed by a 40 s pause; this cycle was repeated once for every 200 μl lysate. Samples were centrifuged at 16100 × g for 10 min at room temperature then filtered with a 100 μm nylon mesh. Supernatants were diluted with 1:1 PBS (v/v) containing protease inhibitors and incubated for 1 h with Dynabeads MyOne Streptavidin C1 (Life Technologies). Beads were washed with lysis buffer, then low-salt buffer (1% Triton X-100, 20 mM Tris (pH 8.0), 2 mM EDTA and 150 mM NaCl), then high-salt buffer (1% Triton X-100, 20 mM Tris (pH 8.0), 2 mM EDTA and 500 mM NaCl), then LiCl salt wash buffer (100 mM Tris (pH 8.0), 500 mM LiCl and 1% IGEPAL® CA-630 (Sigma Aldrich), then twice in lysis buffer.

### Isobaric tags for relative and absolute quantitation (iTRAQ) mass spectrometry analysis

- *Sample preparation:* Following iPOND, beads were resuspended in 30 μl 1 M triethylammonium bicarbonate (TEAB) 2% SDS and incubated for 30 min at 95°C with centrifugation every 10 min at 1000× rpm for 1 min. Suspension was placed on a DynaMag-2 magnetic stand (Invitrogen), supernatant collected and stored at –80^o^C for mass spectrometry or immunoblotting.
- *iTRAQ labelling and peptide fractionation:* 100 μg of protein in 50 μl 1M TEAB was reduced with 0.1 volumes of DTT (50 mM) and alkylated with 0.05 volumes of Iodoacetamine (200 mM). Samples were diluted with 1M TEAB to reduce SDS concentration <0.05% before tryptic digestion (10:1 substrate:enzyme). Peptides were dried and resuspended in 30 μl 1M TEAB and labelled with the appropriate iTRAQ reagent according to the manufacturer's instructions. Prior to LC–MS/MS, peptides were fractionated off-line using high pH reversed phase chromatography (49). The gradient was run at 750 μl/min using initially 99.5% buffer A (0.1% ammonium hydroxide, adjusted to pH 10.5 with formic acid) and 0.5% buffer B (0.1% ammonium hydroxide, 99.9% acetonitrile). After 30 min, buffer B was increased to 50% for 4 min, increased to 75% for 4 min and then reduced down to 0.5%. 30-second fractions were collected then concatenated to produce 24 samples for mass spectrometry and dried in a SpeedVac.
- *Mass spectrometry:* Mass spectrometry was performed using a 6600 TripleTof system (AB SCIEX) attached to an Eksigent 425 NanoLC (Eksigent). Peptides were separated using an Acclaim PepMap 100 C18 column. Buffer A comprised 2% acetonitrile, 0.1% formic acid, 98% water. Buffer B comprised 80% acetonitrile, 0.1% formic acid and 20% water. Lyophilised peptides were resuspended in 10 μl sample buffer (2% v/v acetonitrile and 0.1% v/v formic acid), with 1 μl injected. Peptides were loaded at 5 μl/min for 10 min prior to being eluted over a 120 min gradient at 0.3 μl/min. Samples were acquired in IDA mode, with the iTRAQ collision energy adjustment selected. QC samples in the form of 1 μl injections of 100 fmol PepCalMix (AB SCIEX, USA) were run every five samples. At the beginning and end of the batch, 1 μl of control samples in the form of purified k562 peptides were injected.
- *Data analysis:* Mass spectrometry data was processed by a ‘Thorough’ search against the UniProt Swiss-prot human database using ProteinPilot 5.0.1 software (Paragon version 5.0.1.0, 4874) with default settings including the allowance of one missed or nonspecific cleavage (AB SCIEX), 8 plex iTRAQ fixed modifications, with bias correction. A reverse decoy database was used for FDR estimation. Raw abundances were normalised to parental untreated RKO cells using Microsoft Excel and presented as log_10_ values.

### ADP-ribosylation mass spectrometry

Cells were seeded in 15 cm dishes and treated as described in figure legends before harvest by washing twice with ice-cold PBS and scraping into 2 ml ice-cold PBS per 15 cm dish. Lysate was collected and cells pelleted by centrifuging at 500 g for 3 min at 4°C, with delayed deceleration. Cells were lysed with 12 ml lysis buffer per 15 cm dish (6 M guanidine–HCl, 50 mM Tris pH 8.5), followed by vigorous vortexing and shaking in alternating 5 s intervals for a total of 30 s, before snap freezing in liquid nitrogen and storage at -80°C.

ADP-ribosylated peptides were enriched for mass spectrometry as previously described (35,43,44). Briefly, lysates were thawed at room temperature, treated with 5 mM TCEP and 5 mM CAA (both Sigma Aldrich) for reduction and alkylation, and sonicated on ice for 45 s at 70% amplitude. Proteins were digested with Lys-C endopeptidase (1:400 w/w; Wako Chemicals) for 3 h, diluted to 1.5 M guanidine–HCl with 50 mM ammonium bicarbonate, and incubated overnight with modified sequencing-grade Trypsin (1:400 w/w; Sigma Aldrich). Lys-C and trypsin were inactivated with trifluoroacetic acid (final concentration 0.5% v/v) before high-speed centrifugation to remove precipitates. Peptides were purified using reverse-phase Sep-Pak C18 cartridges (Waters Corp., MA, USA) according to the manufacturer's instructions, eluted in 30% acetonitrile in 0.1% trifluoroacetic acid, and 5% of each sample set aside for proteome analysis. The remaining purified peptides were frozen at least overnight at –80°C, lyophilized for 96 hr, and reconstituted in AP buffer (50 mM Tris–HCl (pH 8.0), 50 mM NaCl, 1 mM MgCl_2_ and 250 μM dithiothreitol), and 4.6 mg peptides aliquoted for ADP-ribose (ADPr) enrichment. ADP-ribose polymers were reduced to monomeric form by overnight incubation with 1:10 000 w/w recombinant hPARG (a kind gift from Prof. Michael O. Höttiger) and gentle shaking, and precipitates removed by centrifuging at 4°C for 30 min at 4250 × g. Peptides were incubated with GST-tagged Af1521 macrodomain beads at 100 μl dry beads/10 mg sample and incubated in a head-over-tail mixer at 4°C for 3 h, before washing twice with ice-cold AP buffer, twice with ice-cold PBS with 250 μM dithiothreitol, and twice with ice-cold water. ADP-ribosylated peptides were eluted with 0.15% trifluoroacetic acid and centrifuged through 0.45 μm spin filters, followed by centrifugation through 100 kDa cut-off spin filters (Vivacon). Peptides were desalted and fractionated using four-layer stage tips at high pH as previously described (35,43,44) and eluted in two fractions with 10% or 15% acetonitrile in 50 mM ammonium hydroxide. Fractionated peptides were vacuum-dried at 60°C and reconstituted in 0.1% formic acid for liquid chromatography and tandem mass spectrometry (MS/MS) analysis.

ADPr mass spectrometry was performed on an Orbitrap Fusion Lumos™ Tribrid™ mass spectrometer (Thermo Fisher Scientific) using electron-transfer/higher-energy collision dissociation fragmentation. Samples were analysed on 15–20 cm analytical columns (internal diameter 75 μm), packed in-house using ReproSil-Pur 120 C18-AQ 1.9 μm beads (Dr. Maisch) connected to a nanoscale EASY-nLC 1200 liquid chromatograph (Thermo Fisher Scientific). Peptides were eluted from the analytical column heated to 40°C in a column oven, with a gradient of buffer A (0.1% formic acid) and buffer B (80% acetonitrile in 0.1% formic acid). For high pH fractions, the primary gradient ranged from 3–38% buffer B over 38 min, then an increase to 90% buffer B over 2 min, constant 90% buffer B for 4 min, decrease to 5% buffer B over 3 min, and 5% buffer B for 3 min. Electrospray ionization was achieved using a NanoSpray Flex NG ion source (Thermo Fisher Scientific; spray voltage 2 kV, capillary temperature 275°C, radio frequency level 40%). Full scans were performed at 120000 resolution, with a scan range of 300–1750 *m*/*z* and maximum injection time at auto. The normalized automatic gain control (AGC) target was 50. Precursor isolation was performed at a width of 1.3 m/z, normalized AGC target of ‘400’, and fragmentation using electron transfer dissociation with supplemental higher-collisional dissociation with a normalized collision energy of 20. Top 3 precursors with charge state 3–5 were isolated for MS/MS analysis, with a dynamic exclusion of 60 s. MS/MS spectra were measured with a maximum precursor injection time of 1000 ms and at 60000 scan resolution.

Raw mass spectrometry data was analysed using MaxQuant software version 1.5.3.30 with default settings unless indicated. A human fasta file downloaded on 11.03.2021 from Uniprot.org, containing an additional *TIMELESS* protein sequence with the ALQR deletion, was used to generate a theoretical spectral library for the database search. The maximum missed cleavages was set to 6. Methionine oxidation, N-terminal acetylation, cysteine carbamidomethylation, and ADP-ribosylation on cysteine, aspartic acid, glutamic acid, histidine, lysine, arginine, serine, threonine, and tyrosine residues were set as variable modifications. The maximum number of modifications per peptide was set to 5. Label-free quantification (LFQ) by fast LFQ was enabled (50) and normalization type set to none. Match between runs was enabled with a match time window of 0.7 min and alignment time window of 20 min. After MaxQuant processing, data were manually filtered with Perseus (51) (MaxQuant) and Microsoft Excel to identify and localize ADP-ribosylation sites as follows: peptide-spectrum matches with more than one ADPr modification were excluded, and only ADPr site assignments with localization probability above 0.9 were included for site identification and above 0.75 for quantification. Because default MaxQuant intensity assignments to modification sites also include intensities from unlocalized or poorly localized evidence, intensity values were manually mapped from the evidence.txt to the sites table based on localized peptide-spectrum matches only.

Intensity values were normalized using the variance stabilization normalization method in the R package VSN (52). Mixed imputation of missing data points was performed by first determining ‘missing not at random’ data points as those that were missing in all replicates of any one condition. Intensity values for these datapoints were imputed as 0. The remaining datapoints were assumed to be ‘missing at random’ and were imputed using a K-nearest neighbor model in the DEP R package (53). To determine differentially PARylated sites, pairwise comparisons were performed using the Limma R package (54), using a Benjamini–Hochberg corrected *P* value of 0.05 as the threshold for significance. An interaction term was also included, which identifies sites differentially ADP-ribosylated between Δ4 and parental cells as a function of time of exposure to PARGi.

### Quantification and statistical analysis

Prism 9 (GraphPad) was used for statistical analysis, where *P< 0.05, **P< 0.01, ***P< 0.001, ****P< 0.0001, ns: *P*> 0.05. Details of statistical analyses are described in the figure legends.

## Results

### An RNAi-complementation assay to probe Timeless function in RKO cells

**Figure 1.**
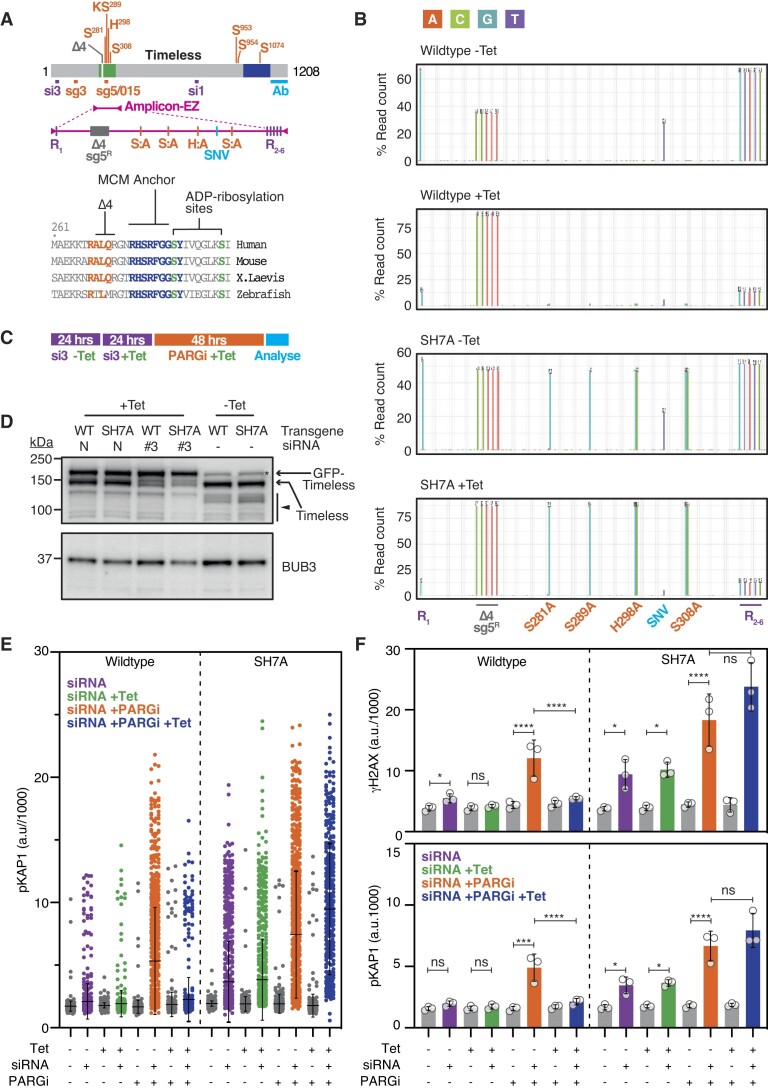
A Timeless SH7A ADP-ribosylation mutant does not restore PARGi-resistance. (**A**) Schematic of Timeless showing locations of the PARP1 binding site (dark blue), seven ADP-ribosylation sites (orange), siRNAs (si1/3, purple), sgRNAs (sg3/5/015, orange) and the anti-Timeless antibody binding site (Ab, light blue). The green box highlights the region analysed by Amplicon-EZ sequencing, showing the location of six RKO-specific nucleotide variants (purple), an allele-specific SNV (light blue), four of the mutated ADP-ribosylation sites (S/H:A, orange). The grey box indicates the location of the Δ4 deletion and overlapping sg5 sequence, with the amino acid sequence expanded below, highlighting the four amino acids deleted in Δ4 (RALQ, orange), the highly conserved MCM-plugin anchor (blue) and two adjacent ADP-ribosylation sites (green). (**B**) Bar graphs comparing *TIMELESS* cDNA read counts in WT and SH7A lines, with and without transgene-induction (500 ng/ml tetracycline (tet), 48 h). Annotations as in (**A**). (**C**) Timeline of RNAi complementation assay used in panels (**D–F**). RKO cells harbouring tet-inducible, RNAi-resistant, GFP-tagged Timeless transgenes (WT or SH7A) were transfected with siRNA #3, or a non-targeting control (N), exposed to 500 ng/ml tetracycline, treated with 1 μM PARGi then analysed. (**D**) Immunoblots showing expression of endogenous and GFP-tagged Timeless following siRNA transfections and tet-induction as indicated. Arrowhead points to Timeless degradation products. BUB3 is used as a loading control. (**E**) Column scatter plots quantitating pan-nuclear pKAP1 signal. Each symbol represents a single cell, with 827–1000 cells analysed per condition. Bars represent the mean ± SD. Representative experiment of the three biological replicates shown in (**F**). Bar graphs quantitating pan-nuclear pKAP1 and γH2AX signal. Values represent the mean ± SD from three biological replicates. One-way ANOVA with Šídák's multiple comparisons test, *P< 0.03, ***P< 0.0002, ****P< 0.0001, ns: *P*> 0.03.

To explore the role of Timeless in PARG inhibitor resistance, we set up an RNAi complementation assay using RKO cells as a model system. Importantly, these cells, which are near-diploid and karyotypically stable (55), genetically tractable (37) and amenable to high-resolution cell cycle analysis (56), are PARG inhibitor-resistant (Supplementary Figure S1a). First, we generated a synthetic cDNA encoding the wildtype Timeless protein but resistant sgRNA sequences, then mutated it to confer resistance to siRNA sequences targeting *TIMELESS* (Figure 1A). This was then GFP-tagged to differentiate it from the endogenous protein, cloned into a T-Rex™ vector to enable tetracycline-controlled expression, and integrated at a pre-defined genomic locus using Flp-In™ mediated recombination. In parallel, we generated an RKO derivative harbouring a Timeless transgene with seven known ADP-ribosylation sites mutated from either serine or histidine to alanine (SH7A, Figure 1A) (32,35,57). Note that it was recently shown that mutating six of these sites does indeed inhibit PARylation of Timeless (32). We also generated Timeless S289A, where only the dominant ADP-ribosylation site was substituted (35,57), and a variant harbouring a four amino acid deletion (Δ4, Figures 1A, S1b), the rationale for which is described below. To verify tetracycline-mediated induction of the appropriate transgenes, Timeless cDNA was analysed using Amplicon-EZ-based next-generation sequencing (NGS) (Figure 1B). While transgenic-specific variants were detected in the absence of tetracycline, indicating leaky expression, the addition of tetracycline markedly increased transgene expression, evidenced by the observation that the allele frequencies of RKO-specific single nucleotide variants (SNVs) fell from >60% to <25%, and the allele frequencies of transgene-specific mutations increased from <40% to ∼80%. In the case of Timeless SH7A, the frequency of the variants encoding the alanine substitutions increased, thus distinguishing it from WT (Figure 1B).

### A Timeless SH7A ADP-ribosylation mutant does not restore PARGi-resistance

To test the ability of ectopic GFP-tagged WT and SH7A proteins to support Timeless function, cells were transfected with siRNAs in the absence of tetracycline to initiate repression of the endogenous protein, then re-transfected in the presence of tetracycline to maintain repression whilst also inducing the transgene (Figure 1C). After exposure to the PARG inhibitor PDD00017273 (hereafter PARGi) for 48 hours, the cells were analysed. Immunoblotting showed that in the absence of tetracycline, GFP-tagged proteins were detectable, confirming leaky expression (Figure 1D). However, in the presence of tetracycline, levels of the GFP-tagged proteins increased and, when combined with siRNA transfection, became more abundant than endogenous Timeless.

Previously, we showed that intrinsically sensitive cell lines exposed to PARGi exhibit markers consistent with persistent DNA replication stress, replication catastrophe and DNA damage, including γH2AX and pKap1 (15,58,59). By contrast, resistant cell lines do not. Thus, in the presence of intact DNA damage response pathways, γH2AX and pKap1 serve as a molecular proxies for PARGi sensitivity. Immunofluorescence analysis of pan-nuclear pKAP1 and γH2AX showed that Timeless RNAi induced replication catastrophe in a small subset of cells (Figure 1E, F, see purple symbols). However, consistent with our previous screen (12), this was dramatically escalated when combined with PARG inhibition (orange). Strikingly, while induction of WT GFP-Timeless largely suppressed PARGi-induced pKAP1 and γH2AX, the SH7A mutant failed to do so (blue). Indeed, even in the absence of tetracycline, the presence of the SH7A mutant due to leaky expression exacerbated pKAP1 and γH2AX staining upon siRNA (purple). Thus, these observations confirm that Timeless is required to tolerate pharmacological inhibition of PARG (12), but also show that this ability is dependent on the presence of several amino acids that are known ADP-ribosylation sites (Figure 1A).

### Targeting *TIMELESS* via CRISPR-Cas9-mediated gene editing in FNE1 cells

**Figure 2.**
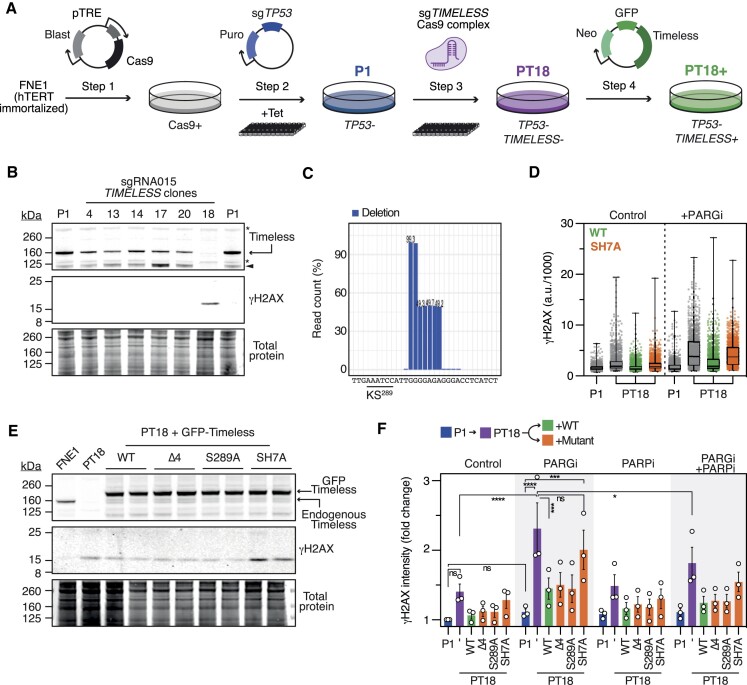
Targeting *TIMELESS* via CRISPR-Cas9-mediated gene editing in FNE1 cells. (**A**) Schematic showing manipulation of FNE1 cells to introduce a tet-inducible Cas9 (step 1) followed by CRISPR-mediated mutation of *TP53* to create P1 cells (step 2) (60). P1 cells were then transiently transfected with an RNP complex consisting of Cas9 and sgRNA015 targeting *TIMELESS*, and single-cell clones expanded to create PT18 cells (step 3). PT18 cells were then transduced to introduce GFP-tagged Timeless transgenes to create PT18+ (step 4). Blast, blasticidin: Neo, neomycin; Puro, puromycin. (**B**) LICOR-based immunoblots showing Timeless and γH2AX levels in PT clones. Total protein stain is shown as a loading control. Asterisks (*) represent non-specific bands, arrowhead points to a Timeless degradation product. (**C**) Bar graph showing read counts of *TIMELESS* gDNA amplicons from PT18 cells. Location of ADP-ribosylation site KS^289^ indicated for reference. (**D**) Column scatter plots quantitating nuclear γH2AX immunofluorescence in P1, PT18 and PT18+ cells treated with 1 μM PARGi for 72 h. Each symbol represents a single cell, with 1000 cells analysed per condition, from one representative experiment. Boxes represent median and interquartile ranges, whiskers represent minimum and maximum values. (**E**) LICOR-based immunoblots showing Timeless and γH2AX levels in FNE1, PT18 and PT18+ populations transduced with two different lentiviral MOI values. Total protein stain is shown as a loading control. (**F**) Bar graph quantitating fold-change in nuclear γH2AX intensity in P1, PT18 and PT18+ cells treated for 72 h with 1 μM PARPi and/or 1 μM PARGi as indicated. Values represent mean values ± SEM from three biological replicates. Two-way ANOVA, *P< 0.05, *** *P*< 0.001, ****P< 0.0001, ns: *P*> 0.05.

To further test the significance of Timeless’ ADP-ribosylation sites, we turned to a different approach, using CRISPR-Cas9-mediated gene editing to mutate *TIMELESS*. Here, we chose FNE1, an immortalized cell line generated by hTERT transduction of non-ciliated fallopian tube epithelial cells, the putative cell of origin for many HGSOCs (41). FNE1 cells are karyotypically stable and largely disomic, except for monosomies at 9p, 15 and X due to loss of the inactive X chromosome and an unbalanced translocation between the short arm of chromosome 9 and chromosome 15 (60,61). FNE1 cells are non-transformed and p53-proficient; therefore to increase the chance of recovering *TIMELESS* mutants, we opted to use a*TP53*-deficient derivative generated previously (FNE1 P1, Figure 2A) (60). P1 cells were transiently transfected with a ribonucleoprotein (RNP) complex consisting of Cas9 and an sgRNA targeting *TIMELESS* (Figure 1A). Single-cell clones were expanded (Figure 2A) and screened by immunoblotting, leading to the identification of one clone, PT18, that lacked detectable Timeless protein (Figure 2B). NGS analysis of *TIMELESS* gDNA amplicons identified a two base pair (bp) deletion approaching 100% frequency, and an additional five bp deletion approaching 50% (Figure 2C). Our interpretation is that the two *TIMELESS* alleles harbour two different but overlapping deletions, one involving two base pairs, the other seven base pairs. Both deletions cause frameshifts shortly followed by STOP codons (not shown), consistent with the lack of detectable Timeless protein (Figure 2B).

### Timeless SH7A does not restore PARGi-resistance in *TIMELESS*-mutant FNE1 cells

Notably, PT18 grew slower than clones that retained Timeless (Supplementary Figure S2a) and had elevated γH2AX (Figures 2B, D and S2b), indicating the presence of DNA damage, possibly due to perturbed DNA replication (28). Moreover, γH2AX escalated dramatically upon exposure to PARGi, consistent with the siRNA-based experiments described above. To confirm that this was due to loss of Timeless function—as opposed to an off-target CRISPR mutation—PT18 cells were transduced with a lentiviral vector encoding GFP-tagged Timeless to create PT18+ (Figure 2A). In parallel, PT18 cells were transduced with Timeless SH7A, S289A and Δ4. Immunoblotting confirmed expression of the various GFP-tagged transgenes, and while the WT, Δ4 and S289A constructs suppressed γH2AX, SH7A clearly enhanced it (Figure 2E). In response to PARG inhibition, immunofluorescence analysis showed that WT, Δ4 and S289A, significantly alleviated PARGi-induced γH2AX (Figures 2D and F). Co-exposure to the PARP1/2 inhibitor Olaparib (hereafter PARPi) also significantly ameliorated the PARGi effect (Figure 2F). By contrast, expression of SH7A failed to restore γH2AX to control levels (Figures 2D and F).

Thus, although genome-wide CRISPR screens suggest that *TIMELESS* is an essential gene (Supplementary Figure S2c), these observations indicate that it is possible to isolate Timeless-deficient cells, at least in the context of *TP53* mutation. In turn, our analysis of PT18 cells further strengthens the conclusion that Timeless is required to tolerate pharmacological inhibition of PARG, and that this ability is dependent on known ADP-ribosylation sites. Although S289 is the predominant ADP-ribosylation site in Timeless (35,57), and is in the context of a preferred ‘KS motif’ (62–64), mutation of this serine alone is not sufficient to phenocopy SH7A, suggesting that multiple substitutions are required to block Timeless modification (32). Because mutation of *TIMELESS* alone elevated basal γH2AX, we were concerned that DNA damage accumulated during the expansion of PT18, coupled with its slow growth kinetics, might complicate its use as a model system. Therefore, as described below, we turned our attention to a *TIMELESS* haploinsufficient model that proliferates with near-normal kinetics but is exquisitely PARGi sensitive.

### Targeting *TIMELESS* via CRISPR-Cas9-mediated gene editing in RKO cells

**Figure 3.**
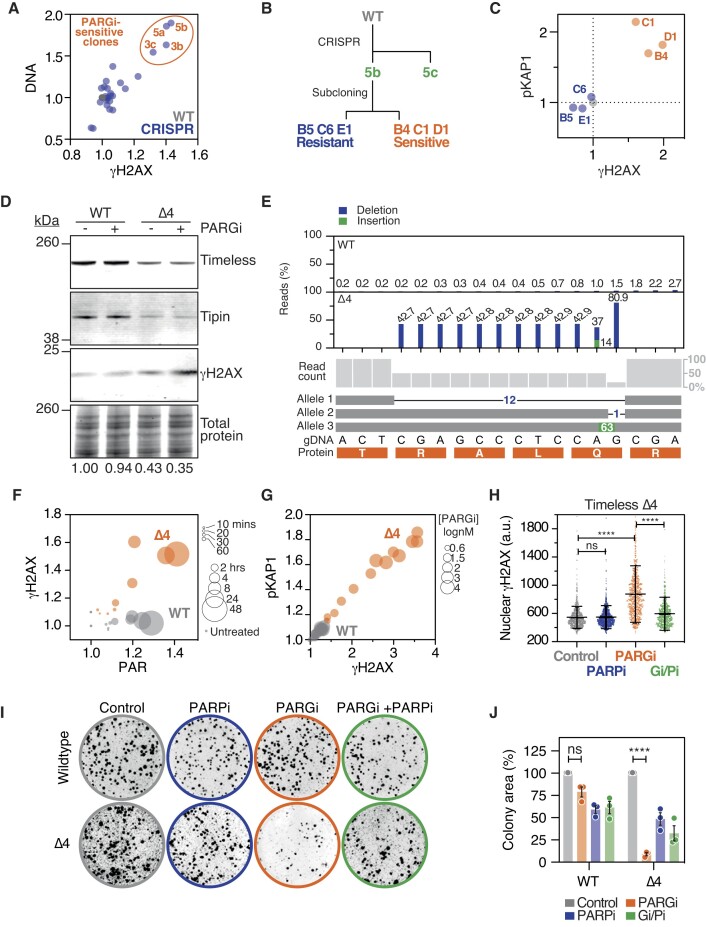
*TIMELESS* Δ4 cells are heavily dependent on PARG activity. (**A**) Scatter plot showing nuclear γH2AX intensity versus total nuclear DNA staining for CRISPR/Cas9 clones (blue) following 72 h 1 μM PARGi treatment. Symbols represent mean values from one experiment, normalized to parental RKO control cells (WT, grey). (**B**) Schematic showing isolation of Timeless Δ4 clone 5b.C1. *TIMELESS* was targeted in parental RKO cells (WT) yielding mixed-population 5b, which then underwent subcloning to yield PARGi-resistant and PARGi-sensitive monoclonal derivatives. 5b.C1 was used for all subsequent analysis. (**C**) Scatterplot showing nuclear pKAP1 and γH2AX levels in the indicated subclones following exposure to 1 μM PARGi for 72 h, normalized to parental RKO (grey). Data from one experiment. (**D**) LICOR-based immunoblot showing Timeless, Tipin and γH2AX levels in WT and Δ4 cells treated with 1 μM PARGi for 24 h. Total protein stain is shown as a loading control. Mean Timeless quantitation from three biological replicates given at the bottom. (**E**) Bar graph showing read counts of *TIMELESS* gDNA amplicons from WT and Δ4 cells, indicating presence of alleles with a 12-nucleotide deletion, a single-nucleotide deletion and a 63-nucleotide insertion. (**F**) Scatter plot quantitating γH2AX and PAR signal in WT (grey) and Δ4 cells (orange) exposed to 1 μM PARGi for the indicated times (bubble size). Values represent mean of three biological replicates normalized to untreated parental control. (**G**) Scatter plot quantitating γH2AX and pKAP1 signal in WT (grey) and Δ4 cells (orange) exposed to PARGi at indicated concentrations (bubble size) for 72 h. Values represent mean of three biological replicates normalized to WT untreated control. (**H**) Column scatter plot quantitating γH2AX signal in Δ4 cells treated for 72 h with 1 μM PARPi (Pi) and/or 1 μM PARGi (Gi). Symbols represent single cells, with 1000 cells analysed per condition. Bars represent the mean ± SD. Representative of three independent experiments. One-way ANOVA, ****P< 0.0001, ns: *P*> 0.05. (**I**) Colony formation of WT or Δ4 cells in the continuous presence of 1 μM PARPi (Pi) and/or 1 μM PARGi (Gi). Representative images of three independent experiments. (**J**) Bar graph quantitating clonogenic potential in the continuous presence of 1 μM inhibitors as indicated, normalized to untreated control. Bars represent mean ± SEM from three independent experiments. Two-way ANOVA, ****P< 0.0001, ns: *P*> 0.05.

In a separate approach, we used CRISPR-Cas9 to mutate *TIMELESS* in RKO cells using sgRNAs targeting N-terminal exons (Figure 1A). While we failed to identify clones lacking Timeless protein (Supplementary Figure S3a), exposure to PARGi identified four potentially sensitive clones, evidenced by pan-nuclear γH2AX and increased DNA content (Figure 3A) (12,15). Focusing on clone 5b, Sanger sequencing of cloned cDNAs identified an in-frame 12 base pair deletion (Supplementary Figure S3b), resulting in a deletion of four amino acids, RALQ, at position 267–270, upstream of a highly conserved motif that forms the MCM-plugin anchor (19), and several ADP-ribosylation sites (Figure 1A). Upon propagation of clone 5b, the proportion of cells displaying markers of sensitivity diminished (not shown), possibly due to outgrowth of wildtype parentals. Indeed, subcloning 5b yielded lines that either did or did not yield a γH2AX/pKAP1 response upon PARG inhibition (Figures 3B, C and Supplementary Figure S3c), with only the γH2AX/pKAP1 positive clones harbouring the 12 bp deletion. Subclone 5b.C1, referred to as ‘Δ4’, was selected for further experiments. Quantitative immunoblotting showed that Δ4 cells expressed lower levels of Timeless and its binding partner Tipin (Figure 3D). NGS analysis of *TIMELESS* gDNA amplicons in Δ4 cells revealed a more complex picture compared with the cDNA analysis; 10 of the 12 affected bases were deleted in ∼43% of amplicons, while the 12th was deleted in ∼80%, and at the site of the 11th affected base, we also detected a large insertion (Figure 3E). Our interpretation of this is that Δ4 cells have three *TIMELESS* alleles with one harbouring a 12-nucleotide deletion that deletes the RALQ sequence, while the second and third alleles harbour a 1-nucleotide deletion and a 63-nucleotide insertion respectively (Figure 3E), resulting in premature STOP codons.

### *TIMELESS* Δ4 cells require PARG activity for proliferation and survival

To characterize Δ4 cells in more detail, we performed a PARGi time-course and drug titration, analysing PAR intensity as a pharmacodynamic biomarker, and pan-nuclear γH2AX and pKAP1 as markers of replication catastrophe. In both WT and Δ4 cells treated with 1 μM PARGi, PAR chain stabilization was evident within 10–60 min and continued to increase for 24–48 h (Figure 3F). While γH2AX levels remained low in WT cells, it increased noticeably in Δ4 cells after 2 hours and continued to increase for the rest of the time course (Figure 3F). Similarly, in response to a drug titration, both γH2AX and pKAP1 remained low in WT cells, even at very high PARGi concentrations (Figure 3G). By contrast, both markers increased in Δ4 cells in a dose-dependent manner, with low nanomolar PARGi concentrations sufficient to induce a notable phenotype (Figure 3G). Thus, PARGi stabilises PAR chains in both WT and Δ4 cells, but while this is tolerated in WT, it results in DNA damage and replication catastrophe in Δ4 cells. Importantly, co-exposure to PARPi markedly ameliorated the PARGi effect in Δ4 cells (Figure 3H), indicating that in this context PARGi toxicity also requires PAR chain assembly. To determine the longer-term consequences of PARGi exposure, WT and Δ4 cells were analysed by colony formation assays. Consistent with the ability of WT cells to tolerate stabilized PAR chains, their clonogenic potential in the presence of PARGi was robust, ∼75% compared with untreated control (Figure 3I, J). By contrast, the clonogenic potential of Δ4 cells was severely compromised, at <10% of the untreated control. Thus, we conclude that the net effect of the *TIMELESS* mutations in Δ4 cells creates a state whereby there is sufficient Timeless function to support DNA replication, and in turn efficient proliferation (Supplementary Figure S3d). However, proliferation of these cells now requires PARG activity.

### PARGi sensitivity of Δ4 cells is due to *TIMELESS* haploinsufficiency

To better understand the PARGi sensitivity of Δ4 cells, we asked whether the 12-base pair deletion alters Timeless function. One possibility is that the four amino acid deletion has little or no functional consequence. In which case, PARGi sensitivity of Δ4 cells may simply be due to haploinsufficiency, i.e. reduced Timeless expression due to inactivation of the other two alleles. However, the isolation of PT18 suggests that cells can survive complete loss of Timeless; therefore, a second possibility is that ΔRALQ creates a hypomorph, and that PARGi-sensitivity arises due to the combined effect of reduced expression due to two null alleles *and* compromised biochemical function of the remaining Δ4 protein. Indeed, because we did not identify RKO or FNE1 lines heterozygous for *TIMELESS*, i.e. a wild type allele in combination with a null, haploinsufficiency may not sufficiently reduce Timeless function to expose PARG-dependency. To distinguish between these possibilities, we predicted that if the Δ4 phenotype was due only to haploinsufficiency, overexpression of Δ4 should, like WT, restore PARGi-resistance. By contrast, if Δ4 protein is biochemically compromised, it should be unable to restore PARGi-resistance. Consistent with the first prediction, overexpression of Timeless Δ4 rescued RNAi-mediated inhibition of Timeless (Supplementary Figure S1c, d). Similarly, transduction of PT18 cells with a Timeless Δ4 lentivirus alleviated PARGi-induced γH2AX as efficiently as the WT transgene (Figure 2F). This suggests that the Timeless ΔRALQ mutant is largely functional, thus we conclude that the PARGi-sensitivity of Δ4 cells is primarily due to *TIMELESS* haploinsufficiency.

### Pharmacology profiling shows similarity between siTimeless and Δ4 phenotypes

**Figure 4.**
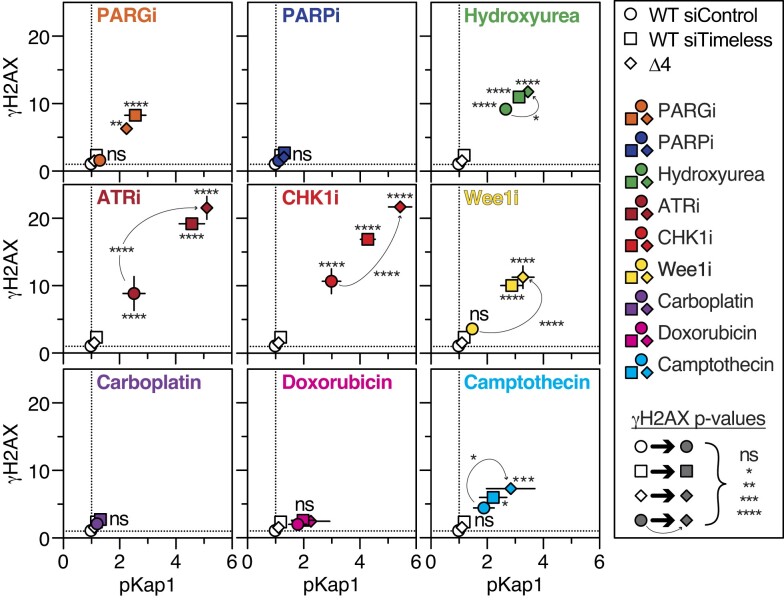
Pharmacology profiling shows similarity between siTimeless and Δ4 phenotypes. XY plots of γH2AX and pKAP1 signal in WT and Δ4 cells following 24 h exposure to the following drugs: PARGi 1 μM, PARPi 5 μM, Hydroxyurea 200 μM, ATRi 50 nM, CHK1i 50 nM, Wee1i 0.5 μM, Carboplatin 15 μM, Doxorubicin 10 nM, Camptothecin 25 nM. Prior to drug exposure, WT cells were transfected with siRNAs, either targeting *TIMELESS* (siTimeless) or a non-targeting control (siControl). Symbols show means ± SEM, normalized to their respective controls derived from at least two biological replicates. Significant comparisons shown (two-way ANOVA, Dunnett's multiple comparisons test), *P< 0.05, **P< 0.01, ***P< 0.001, ****P< 0.0001, ns: *P*> 0.05.

To further test this conclusion, we compared RKO Δ4 cells with siTimeless RKO cells in response to pharmacological agents that interfere with DNA replication and/or the replication stress response (RSR). Consistent with earlier observations (Figures 1E, F and 2D, F), both modes of Timeless inhibition resulted in PARGi-induced γH2AX (Figure 4). By contrast, PARPi did not induce γH2AX in siTimeless or Δ4 cells. Regarding RSR kinases, at the concentrations used, ATRi and CHK1i, but not Wee1i, resulted in modest induction of γH2AX in the parental RKO cells; nevertheless in all three cases, both modes of Timeless inhibition exacerbated γH2AX. By contrast, the alkylating agent carboplatin, or the topoisomerase II inhibitor doxorubicin did not give rise to γH2AX. We also only saw a modest effect with camptothecin, a topoisomerase I inhibitor. Interestingly, there was modest induction of γH2AX in RKO cells with hydroxyurea, a ribonucleotide reductase inhibitor that suppresses deoxyribonucleotide production, and again, both siTimeless and Δ4 exacerbated this. Thus, inhibition of Timeless noticeably induced γH2AX in response to PARGi, the RSR kinase inhibitors and hydroxyurea, but not to PARPi or drugs that directly target the DNA double helix. Interestingly, PARPi can also target the DNA double helix by trapping PARP1/2 at single strand breaks (65,66).

In summary, taking the observation that overexpression of the Δ4 transgene rescues both siTimeless and CRISPR-generated *TIMELESS* mutants, along with the very close alignment of the phenotypes of siTimeless and Δ4 RKO cells, we conclude that the Δ4 phenotype is largely driven by haploinsufficiency. As such the RKO Δ4 cell line provides a tractable model system to study the role of Timeless in PARGi-sensitivity. In contrast to siRNA experiments, it is a stably transfected, clonal cell line, thereby facilitating larger scale and longer-term experiments (see below). In contrast to the PT18 cells (Supplementary Figure S2a), the Δ4 cell line proliferates with near-normal kinetics (Supplementary Figure S3c) and has not gone through a phase of clonal expansion in the complete absence of Timeless, which may have resulted in the accumulation of additional mutations. For simplicity, hereafter, we refer to this haploinsufficient cell line as Timeless Δ4, and parental RKO cells as WT.

### *TIMELESS* Δ4 reduces replisome speed

**Figure 5.**
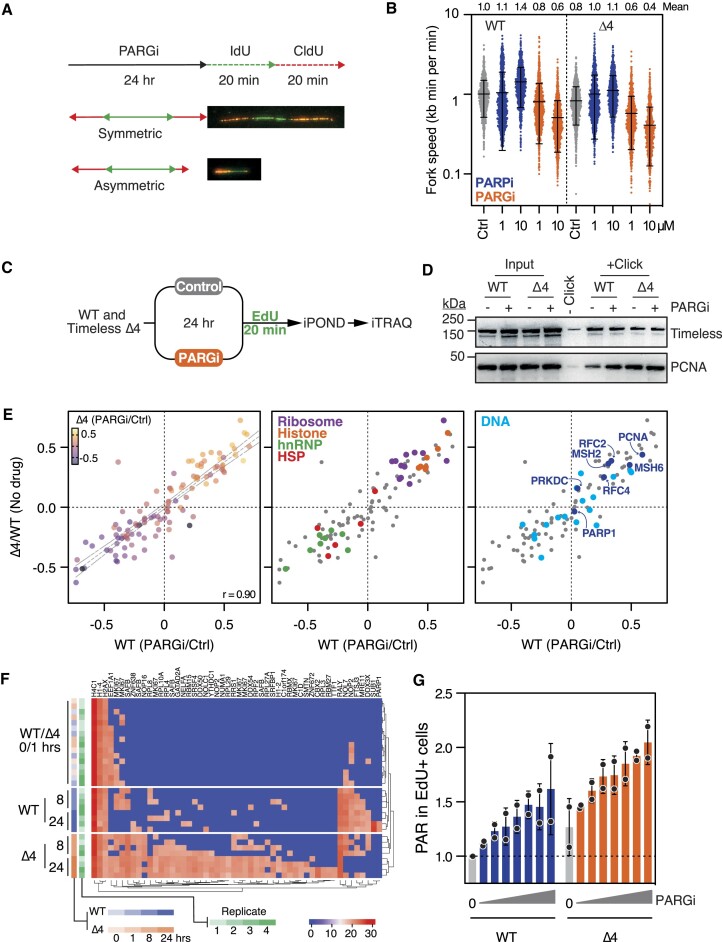
Analysis of replisome speed, composition and the ADP-ribosylome. (**A**) Schematic of nucleotide pulsing experiment to analyse DNA replication fibers, with examples of symmetric and asymmetric forks. Fiber length is used as a proxy for fork speed. (**B**) Column scatter plot quantitating fork speed in RKO WT and Δ4 cells following a 24 h exposure to PARPi or PARGi at indicated concentrations. Each symbol represents a single fiber, with ≥822 measured per condition. Bars show mean ± SD of data from one experiment. Values along top of graph indicate the mean speed from two independent replicates (note: 5B and 6A share controls as they are from the same two experiments). (**C**) Schematic of experiment for *isolation of proteins on nascent DNA* (iPOND) and subsequent analysis by *isobaric tags for relative and absolute quantitation* (iTRAQ). Cells were exposed to 1 μM PARGi. (**D**) Immunoblot of iPOND samples showing input, minus EdU control (-Click) and iPOND-bound samples (+Click), with and without PARGi treatment. (**E**) XY scatter correlations showing fold-enrichment of proteins purified by iPOND, with WT cells treated with PARGi on the *x*-axis and Δ4 cells without treatment on the *y*-axis. *r* = Pearson's correlation. The different graphs highlight (left) relative fold-enrichment from Δ4 cells treated with PARGi; (middle) ribosomal (purple), histone (orange), heterogeneous nuclear ribonucleoproteins (hnRNP; green), and heat-shock proteins (HSP; red); and (right) proteins involved in DNA processes (blue), with factors described in text highlighted (dark blue). Data are from one experiment with two biological replicates per condition. (**F**) Heat map showing clustering of 52 significantly differentially ADP-ribosylated sites across 43 unique proteins identified by pairwise comparison of Δ4 versus WT exposed to PARGi for 24 h. *P*< 0.05, Benjamini–Hochberg method. The other time points (0, 1, 8 h) are also shown, with the four replicates shown separately. (**G**) Bar graph quantitating nuclear PAR signal after a 20-min incubation with 0.1–20 μM PARGi, followed by a 20-min EdU pulse to identify S-phase cells. Bars show the mean ± SD from three independent replicates, with values normalized to WT in the absence of PARGi.

To better understand PARGi sensitivity of Timeless Δ4 cells, we analysed DNA replication fibers by pulsing nucleotide analogues (Figure 5A). Note that we previously showed that PARGi caused fork asymmetry in sensitive cells (12,15), suggesting widespread fork stalling and consistent with the role of PARG in reversing PARP1/2-mediated inhibition of RecQ1-dependent fork re-start (67,68). Fork asymmetry was comparable in untreated WT and Δ4 cells (13%) but increased upon exposure to PARGi (Supplementary Figure S4a). This increase was more pronounced in Δ4 cells, increasing from 29% in WT to 42%. This initial analysis also suggested that fiber lengths were shorter in Δ4 and PARGi-treated cells: therefore, we analysed fork speed in more detail. Consistent with previous observations (69–72), in WT cells PARPi accelerated fork speed in a dose-dependent manner, from an average of 1.0 kb per min to 1.1–1.4 kb/min (Figure 5b). Strikingly, and conversely, PARGi reduced fork speed to 0.6–0.8 kb/min, supporting the notion that opposing PARP and PARG activities control replisome speed. Notably, fork speed was slower in untreated Δ4 versus untreated WT cells (0.8 vs 1.0 kb per min), which was corrected by PARPi (1.0–1.1 kb/min) and exacerbated by PARGi (0.4–0.6 kb/min). One possibility is that to buffer reduced fork speed, S-phase fidelity is maintained by increased origin firing (73–75). Consistently, the number of EdU-positive foci – an indicator of active origins (76,77) – increased in Δ4 versus WT and was further elevated by PARGi (Supplementary Figure S4b). EdU foci trended upwards in PARGi-treated WT, but the increase was not statistically significant. Paradoxically, WT cells can tolerate 0.6 kb/min fork speed, resulting from 10 μM PARGi, yet Δ4 cells cannot tolerate 1 μM, which results in a similar average speed (Figures 5B and S4c). Thus, despite increased origin firing, Δ4 cells still cannot tolerate PARG inhibition, suggesting that the global effect on fork speed alone may not be sufficient to account for PARGi toxicity. We revisit this in the discussion.

### Biochemical analysis of replisomes in *TIMELESS* Δ4 cells

To examine replisome composition, replicating DNA was labelled with EdU followed by *isolation of proteins on nascent DNA* (iPOND; Figure 5C). Immunoblotting showed the presence of Δ4 at active forks (Figure 5D), consistent with its ability to support DNA replication. Interestingly, the DNA sliding clamp PCNA was enriched by both the Δ4 mutation and PARGi exposure (Figure 5D). To facilitate an unbiased analysis of proteins captured by iPOND, we performed quantitative mass spectrometry using *isobaric tags for relative and absolute quantitation* (iTRAQ; Figure 5C; Supplementary Table S3). Consistent with the immunoblotting, PCNA and its binding partners RFC2 and RFC4 were enriched in Δ4 versus WT and in response to PARGi (Figure 5E). Because the EdU pulses were of equal duration in all conditions, coupled with slower fork speed in Δ4 and PARGi-treated cells, the simplest explanation for elevated PCNA is an increased number of active origins, as suggested by the increase in EdU foci. Thus, we conclude that replisome speed is reduced in Timeless Δ4 cells and that this is compensated for by an increase in origin firing (75), thereby sustaining efficient S-phase and cell proliferation. We also observed an increase in the DNA mismatch repair factors, MSH2 and MSH6; this could simply reflect the increase in replisomes or could indicate an increase in stalled forks (78,79). Indeed, we also detected elevated levels of PRKDC/DNA-PKcs that, along with the Ku70/80 heterodimer, form the non-homologous end joining (NHEJ) kinase DNA-PK, recently shown to promote fork reversal and slowing in response to replication stress (80).

### Analysis of the ADP-ribosylome

In a parallel approach, we analysed the impact of Δ4 and PARGi on the ADP-ribosylome (35,43,44). WT and Δ4 cells were exposed to PARGi for zero (*T*_0_), one, eight and 24 h, proteins extracted, and ADP-ribose polymers reduced *in vitro* by treatment with recombinant PARG. Mono-ADP-ribosylated proteins were then isolated via binding to the macrodomain Af1521 and analysed by mass spectrometry. In total, 571 ADP-ribosylation acceptor sites were identified across 304 proteins. 86.2% of modifications occurred on serines, accounting for 99.2% of ADP-ribosylation intensity (Supplementary Figure S5a), corroborating the dominance of serine acceptor sites during replication stress (64). We did not identify Timeless itself, presumably due to low abundance combined with the stochasticity of mass spectrometry. Comparing WT with Δ4 cells at *T*_0_ or after one hour of PARGi exposure did not identify any differentially ADP-ribosylated sites. However, by 8 h, the number of ADP-ribosylated sites in both WT and Δ4 cells approximately doubled (Supplementary Figure S5b) and comparing 0 with 24 h identified more than 200 sites with differential intensity (Supplementary Figure S5c). Indeed, most of the differential ADP-ribosylation occurred between 0 and 24 h, and was common to both WT and Δ4, with only 20 sites on 13 proteins differing significantly when comparing WT versus Δ4 over the time course (Supplementary Figure S5c, d). Consistently, principal component analysis resolved the two latter timepoints from the early timepoints in both cell lines (Supplementary Figure S5e). However, comparing the two lines directly at the 24 h timepoint identified 52 acceptor sites with differential intensity, and unsupervised hierarchical clustering of these sites clearly separates Δ4 from WT cells following eight and 24-hour treatment, with Δ4 cells displaying greater intensity (Figure 5F). The affected sites were on proteins involved in chromatin organization and RNA biogenesis, including MKI67, SAFB and NOP2 (Supplementary Figure S5d). While the significance of this is unclear, it may reflect long-term adaptation to replication stress, for example (81). Thus, prolonged exposure to PARGi induces a subset of proteins to become more heavily ADP-ribosylated in Δ4 cells. This prompted us to ask whether there are also more ADP-ribose polymers in Δ4 cells. Indeed, immunofluorescence analysis of S-phase cells indicated that, across a range of PARGi concentrations, PAR staining was more intense in Δ4 cells compared with WT (Figures 3F and 5G). Even in the absence of PARGi, PAR staining was noticeably higher, suggesting that Δ4 cells are shifted to hyper-PARylated state, possibly due to compromised Timeless function leading to a DNA replication vulnerability that requires PARP1/2 activation to be resolved.

### Nucleoside supplementation rescues PARGi sensitivity in Δ4 cells

**Figure 6.**
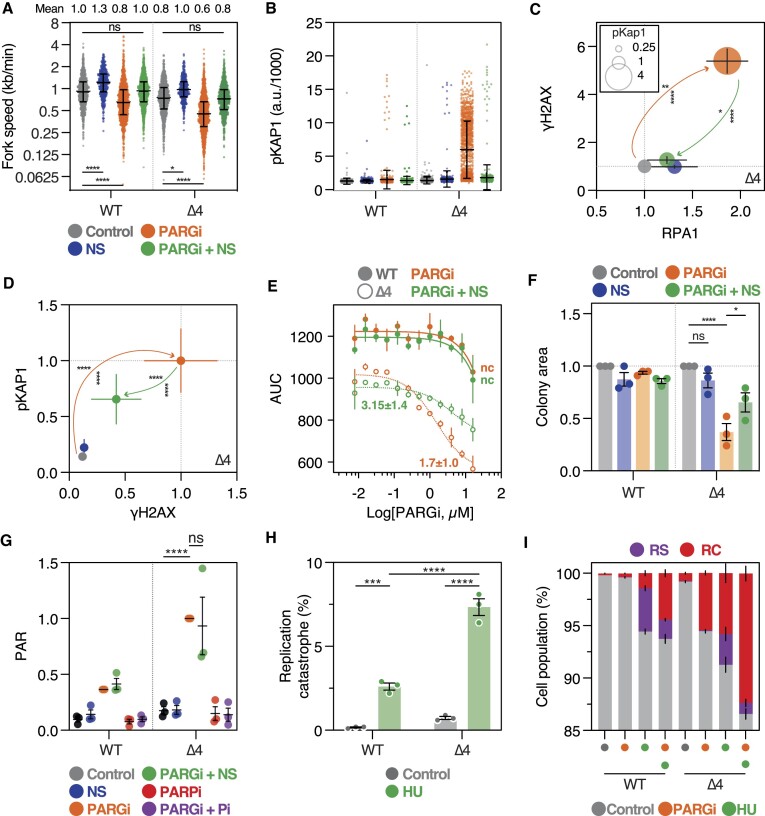
Nucleoside supplementation rescues PARGi sensitivity in *TIMELESS* Δ4 cells. (**A**) Column scatter plot quantitating fork speed in RKO WT and Δ4 cells following 24 h exposures to PARGi and/or nucleoside supplementation (NS, 0.1 mM). Each symbol represents a single fork, with ≥421 measured per condition. Bars show median and 25/75 quartiles. The data shown are from one biological replicate, while the values along the top show the mean from two independent experiments (note: 5B and 6A share controls as they are from the same two experiments). (**B**) Column scatter plot quantitating pKAP1 signal following a 72 h exposure to the treatments indicated by colour scheme in (**A**). Symbols represent single cells, 1000 per condition, and lines represent the mean ± SD. (**C**) XY bubble plot showing quantitation of γH2AX, RPA1 and pKAP1, in Δ4 cells treated for 72 h as per colour scheme in (**A**). Values show the mean ± SEM of multiple biological replicates (*n* ≥ 3), normalized to control. Two-way ANOVA with Tukey's post-hoc test, **** *P*< 0.0001, ** *P*< 0.01, * *P*< 0.05. Statistics for γH2AX and RPA1 shown; pKAP1: *P*< 0.0001 for control versus PARGi and PARGi + NS versus PARGi. (**D**) XY plot showing LiCOR-based quantitation of pKAP1 and γH2AX in Δ4 cells treated for 72 h with the indicated treatments, adjusted for total protein stain and normalised to PARGi-treated Δ4 cells. Values show mean ± SEM from three biological replicates. Two-way ANOVA with Tukey's post-hoc test, **** *P*< 0.0001. Error bars represent SE. (**E**) Line graphs showing area under the curve (AUC) in response to increasing PARGi concentrations ± NS, with AUC values derived from proliferation curves generated by timelapse analysis of single cells over 120 h. Values show mean ± SEM from multiple biological replicates (*n* ≥ 2). Calculated GI_50_ values show mean ± SEM or non-calculable (nc). (**F**) Bar graph showing normalized colony area in response to the continuous treatments indicated. Bars shown mean ± SEM from three biological replicates. One-way ANOVA with Šídák's post-hoc test, ****P< 0.0001, *P< 0.05 ns: *P*> 0.05. (**G**) Column scatter plot showing LiCOR-based quantitation of PAR following 72 h treatments, adjusted for total protein, and normalized to PARGi-treated Δ4 cells. Two-way ANOVA analysis with Tukey's post-hoc test, **** *P*< 0.0001, ns: *P*> 0.05. Mean of three biological replicates. (**H**) Bar graph showing percentage of cells exhibiting replication catastrophe 24 h after a 2 h exposure to 500 μM hydroxyurea (HU), based on pKAP1 and γH2AX signals. Values show mean ± SEM from three biological replicates ± SEM. Two-way ANOVA analysis with Tukey's post-hoc test, ****P< 0.0001, ***P< 0.001. (**I**) Bar graph showing percentage of cells exhibiting replication stress (RS) or replication catastrophe (RC) after a 2 h exposure to 350 μM hydroxyurea (HU) followed by a 24 h low-dose exposure to PARGi (0.25 μM), based on pKAP1 and γH2AX signals. Values show mean ± SEM from three biological replicates.

*In toto*, the analysis of Δ4 cells indicates that PARG inhibition results in persistent DNA replication stress. In various contexts, nucleoside supplementation (NS) has been shown to alleviate DNA replication stress (82,83), so we asked whether NS could restore PARGi resistance in Δ4 cells. First, we analysed DNA fibers; as above, PARGi increased fork asymmetry more markedly in Δ4, and reduced fork speed, in both WT and Δ4 cells (Figure 6A and S6a). In contrast, NS accelerated fork speed in both lines, albeit more markedly in WT vs Δ4. Importantly, however, NS restored fork speed in response to PARGi, with the mean track length similar to that observed in untreated cells. NS also ameliorated PARGi-induced fork asymmetry (Supplementary Figure S6a).

NS also had a dramatic effect on DNA replication stress/catastrophe markers. Single-cell analysis showed that NS almost completely abolished pan-nuclear pKap1 and γH2AX staining in PARGi-treated Δ4 cells, and reduced RPA1 intensity to basal levels (Figure 6B and S7a, b). This reversal was highly significant across multiple biological repeats (Figure 6C and S7c) and was confirmed in population-based immunoblotting experiments (Figure 6d and S7d). The ability of NS to suppress replication stress markers was accompanied by enhanced proliferation. While WT cells were largely impervious to PARGi in a 3-day proliferation assay, Δ4 cells were sensitive with an EC_50_ of ∼1.7 μM (Figure 6E). NS increased the EC_50_ value to ∼3.2 μM (Figure 6E). This in turn correlated with enhanced clonogenic survival. While WT cells were again largely impervious to escalating PARGi concentrations in a colony formation assay, colony area in Δ4 conditions was significantly reduced in a dose-dependent manner (Figures 6F and Supplementary Figure S7e,f). Importantly, NS significantly restored colony area in PARGi-treated Δ4 cells.

Because inhibiting PARP1/2 using PARPi ameliorates PARGi sensitivity, in both intrinsically resistant (12) and Δ4 cells (Figure 3h), one possible explanation for the ability of NS to restore PARGi resistance is inhibition of PARP1/2 (84,85), and is thus simply mimicking PARPi. To test this, we analysed the effect of NS on PAR staining. Consistent with earlier results, PARGi elevated PAR staining, and again this was more marked in Δ4 cells (Figures 6G, S6b). However, while co-exposure with PARPi completely abolished PAR staining, NS did not. Thus, we conclude that the ability of NS to revert PARGi sensitivity is not simply due to inhibition of PARP1/2.

When comparing siTimeless with Δ4, we showed that inhibition of Timeless also induces γH2AX in response to hydroxyurea (Figure 4). In light of NS reversing PARGi sensitivity, we asked whether hydroxyurea might exacerbate PARGi. To test this, measuring pan-nuclear pKap1 and γH2AX, we first confirmed that hydroxyurea induces replication catastrophe more potently in Δ4 cells (Figure 6H). Then we analysed the effect of the combination but, in this case, we differentiated γH2AX foci from pan-nuclear staining to classify cells as either experiencing replication stress (RS) or replication catastrophe (RC) (59). In WT cells, while PARGi had minimal effect, hydroxyurea induced some RS but little RC, and while the combination had little additive effect, it shifted the balance towards RC (Figure 6I). In Δ4 cells, PARGi and hydroxyurea alone induced RC and the combination had a noticeable additive effect, doubling the frequency of cells exhibiting RC.

Thus, we conclude that NS very effectively restores PARGi-resistance in Δ4 cells, evidenced by accelerating fork speed, reducing fork asymmetry, abolishing pKap1 and γH2AX signals, in turn enhancing proliferation and clonogenic potential. Consistent with NS resisting PARG inhibition, an intervention designed to do the opposite, i.e. reducing nucleotide pools via hydroxyurea-mediated inhibition of ribonucleotide reductase, exacerbates PARGi-sensitivity more potently when Timeless function is compromised (Figure 6I).

### Nucleoside supplementation rescues intrinsic PARGi-sensitivity

**Figure 7.**
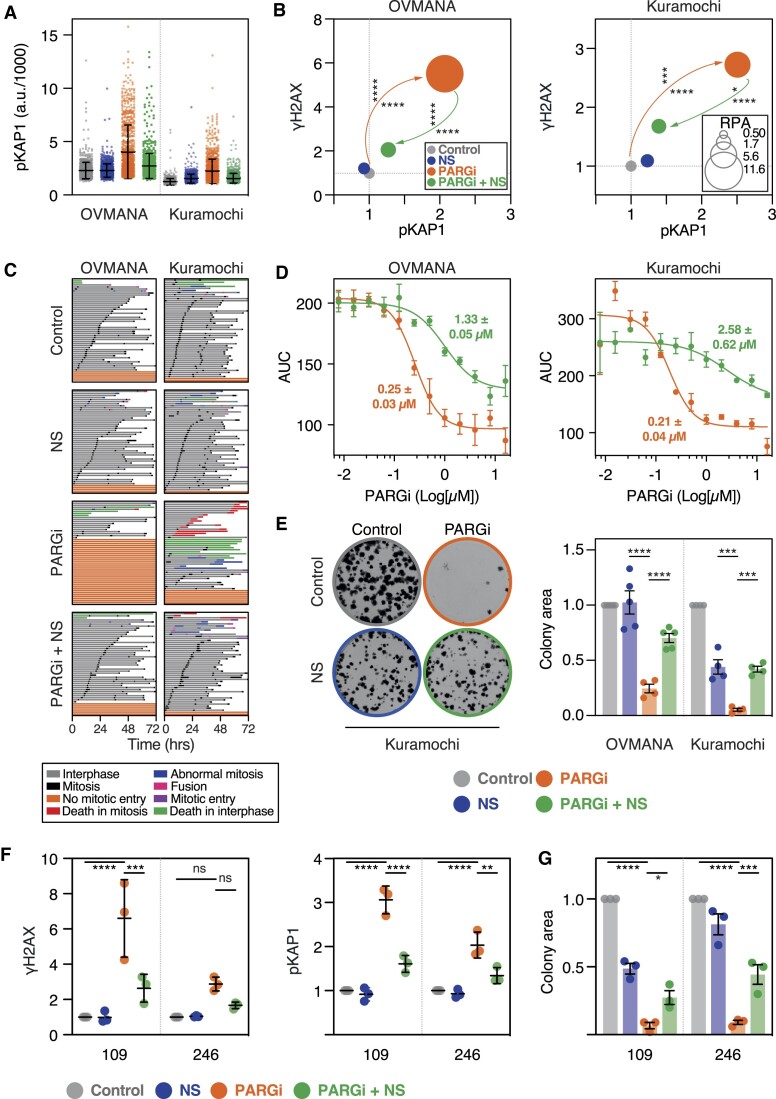
Nucleoside supplementation rescues intrinsic PARGi sensitivity. (**A**) Column scatter plots quantitating pKAP1 in OVMANA and Kuramochi cells following 72 h exposures as indicated by colour scheme: grey, control; blue, nucleoside supplementation (NS); orange, PARGi; green, PARGi + NS. Values show single cells, analysing ≥762 per condition, and bars show the mean ± SD derived from one experiment. (**B**) XY bubble plots of pKap1, γH2AX foci and RPA1 foci (bubble size) following 72 h treatments. Symbols show the mean from multiple biological replicates (*n* ≥ 3), normalized to control. Two-way ANOVA with Tukey's post-hoc test performed, **** P< 0.0001, *** P< 0.001, * P< 0.05. (**C**) Cell fate profiles derived from 72 h time-lapse microscopy analyses with continuous treatments as indicated, after 48 h prior pre-exposure. Horizontal lines show the behaviour of 50 single cells. Representative experiment of two biological replicates. (**D**) Line graphs showing area under the curve (AUC) in response to increasing PARGi concentrations ± 0.25 mM NS, with AUC values derived from proliferation curves generated by timelapse analysis of single cells over 120 h. Values show mean ± SEM from three biological replicates. Calculated GI_50_ values show mean ± SEM. (**E**) Images and bar graphs derived from colony formation assays performed in the continuous presence of indicated treatments (NS 0.25 mM; PARGi 1 μM and 0.5 μM for OVMANA and Kuramochi, respectively). Left: Representative images of Kuramochi. Right: Normalized colony area, with bars showing mean ± SEM percentages of multiple biological replicates (n ≥ 4). One-way ANOVA with Šídák's post-hoc test, ****P< 0.0001, ***P< 0.001. (**F**) Column scatter plots quantitating nuclear γH2AX and pKAP1 intensity in patient-derived ovarian cancer models (OCM) 109 and 246 following 72 h exposures as indicated. Lines represent mean and SD of three biological replicates. Two-way ANOVA with Tukey's post hoc test performed, **** *P*< 0.0001, *** *P*< 0.001, ** *P*< 0.01, ns: *P*> 0.05. (**G**) Bar graph showing normalized colony area for OCMs 109 and 246 following continuous exposures indicated (NS 0.25 mM; PARGi 0.5 μM). Values show mean ± SEM of three biological replicates. One-way ANOVA with Šídák's post-hoc test performed, ****P< 0.0001, ***P< 0.001, * P< 0.05.

The striking ability of NS to rescue PARGi sensitivity in Timeless Δ4 cells prompted us to ask whether it would have a similar effect on intrinsically sensitive models. First, we focused on OVMANA and Kuramochi, established cell lines derived from clear cell and HGSOC respectively (36), which we previously showed are PARGi sensitive (12,15). Single-cell analysis showed that in response to PARGi, NS markedly reduced various RS/RC markers including pKap1, γH2AX and RPA1 (Figures 7A and S8a). NS also reduced γH2AX foci and reversed the mean nuclear area increase that accompanies PARGi-treatment (Supplementary Figure S8a). Again, this reversal was highly significant across multiple biological repeats (Figures 7B and S7c) and was confirmed in population-based immunoblotting experiments (Supplementary Figure S8b, c). As with Δ4, the NS effect was not simply due to inhibition of PARP1/2 as it did not mimic PARPi (Supplementary Figure S8b, d). Timelapse microscopy to analyse cell behaviour over a 72-h period confirmed that inhibition of PARG markedly induced a pre-mitotic block and apoptosis in OVMANA and Kuramochi, respectively (Figure 7C). Both phenotypes were alleviated by NS, and in short-term proliferation assays, NS increased the PARGi EC_50_ values from ∼0.3 μM and ∼0.2 μM to ∼1.3 μM and ∼2.6 μM (Figure 7D). NS also significantly restored clonogenic potential in both cell lines (Figures 7E and Supplementary Figure S8e). Interestingly, NS did not reverse PARGi-induced suppression of colony formation in response to inhibitors of the RSR kinases, carboplatin or hydroxyurea (Supplementary Figure S8f) (86).

Next, we turned to two patient-derived ovarian cancer models, OCM.109 and OCM.246, which are derived from HGSOC and are PARGi-sensitive (15). In both models, NS reduced PARGi-induced increases in γH2AX and pKap1 (Figure 7F). Note the γH2AX effect was modest in OCM.246 and thus the NS effect was not statistically significant. But, nevertheless, the direction of the change was consistent with NS reversing the effect of PARGi. Indeed, NS significantly restored clonogenic potential in both OCM.109 and OCM.246 (Figure 7G). Collectively, these results are striking, in five different model systems, one genetically engineered to be PARGi-sensitive (RKO Δ4), two established cell lines (OVMANA and Kuramochi), and two patient-derived models (OCMs 109 and 246), NS very effectively restores PARGi-resistance.

### Inhibition of thymidylate synthase induces PARGi-sensitivity

**Figure 8.**
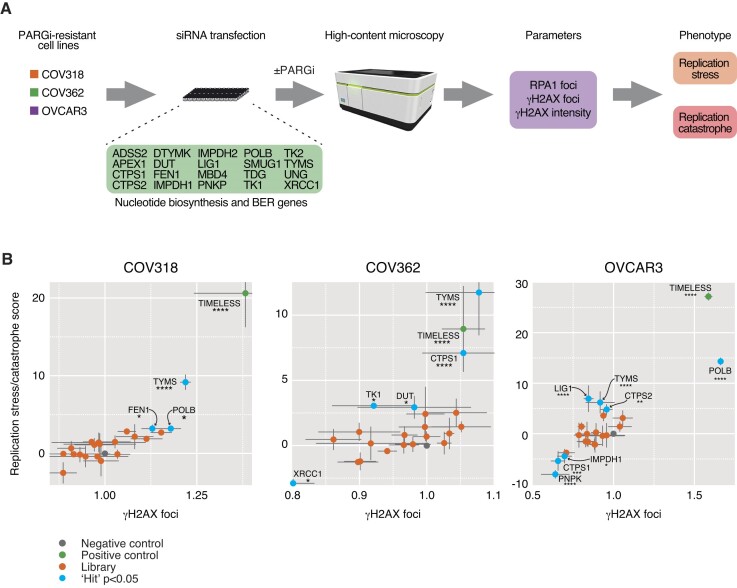
Identification of *TYMS* as a PARGi sensitizer. (**A**) Schematic showing siRNA mini-screen workflow whereby PARGi-resistant cell lines were transfected with siRNAs for 72 h, exposed to 1 μM PARGi for the final 48 h, followed by high-content microscopy, using RPA1 and γH2AX signals to quantitate cells exhibiting replication stress or replication catastrophe. (**B**) XY plots of γH2AX foci versus replication stress/catastrophe score. Negative (grey) and positive controls (siTimeless, green) are indicated. Values show mean ± SEM from at least two biological replicates, with statistically significant values highlighted. Dunnett's multiple comparisons test, significance indicated for replication stress/catastrophe score ****P< 0.0001, **P< 0.01, * *P*< 0.05.

The ability of NS and hydroxyurea to rescue and exacerbate PARGi sensitivity respectively suggests that intrinsic PARGi sensitivity may arise due to compromised nucleotide availability. If this is the case, then inhibiting nucleotide biosynthesis genes in PARGi-resistant cells may confer sensitivity. Also, because nucleotide imbalance can lead to base misincorporation during S-phase (87), in turn increasing the demand on base excision repair (BER) mechanisms, we postulated that inhibition of BER genes may also confer PARGi sensitivity. To test these two ideas, we performed an siRNA mini-screen, focusing on three established HGSOC cell lines that we previously showed to be PARGi resistant, namely COV318, COV362 and OVCAR3 (12,15). These three lines were transfected with a panel of siRNAs targeting 20 genes involved in nucleotide biosynthesis and BER (Supplementary Table S2), exposed to PARGi then analysed by high-content imaging to measure multiple parameters that were then interrogated to generate a RS/RC score (Figure 8A). siRNAs targeting *TIMELESS* were included as an internal positive control. To visualize the results, the RS/RC score was plotted against the number of γH2AX foci (Figure 8B). While the statistically significant hits varied across the three lines, siTimeless potently gave rise to PARGi-induced γH2AX in all three cell lines, consistent with our previous observations (12). The only other statistically significant hit in all three was *TYMS* which encodes thymidylate synthase, the enzyme required for *de novo* production of dTMP (88), a precursor required for dTTP production. Importantly, inhibiting thymidylate synthase not only depletes dTTP pools, but also disrupts production of the other dNTPs due to feedback mechanisms (89). Several other genes emerged as possible PARGi sensitizers, including *CTPS2*, *TK1*, *DUT* (pyrimidine biosynthesis genes) as well as *POLB*, *FEN1*, *LIG1* (BER genes), although their effects were more modest and/or limited to one or two cell lines, highlighting that there may be cell-line-specific redundancies and different buffering capacities. However, *TYMS*, *DUT*, *POLB*, *LIG1* and *FEN1* have been identified by others as PARGi synthetic lethality genes (90,91), and these observations support the notion that inhibition of nucleotide biosynthesis and BER can sensitize cancer cells to pharmacological PARG inhibition.

## Discussion

### A multifaceted approach to defining the determinants of PARG inhibitor sensitivity

To better understand the molecular determinants of PARG inhibitor sensitivity, we took a multifaceted approach, analysing (a) model systems engineered to be PARGi sensitive via inhibition of *TIMELESS*, and (b) intrinsically sensitive ovarian cancer cells. Our key findings are as follows. (i) We extend our previous analysis of ovarian cancer cell lines (12,15) and show that inhibition of Timeless in RKO and FNE1 cells induces PARGi sensitivity, via a mechanism that leads to DNA replication catastrophe. This supports our prior conclusion that sensitivity to PARGi monotherapy results from a DNA replication rather than metabolic vulnerability (15). (ii) In two complementation assays, a Timeless mutant lacking seven ADP-ribosylation sites fails to restore PARGi resistance. (iii) Although complete loss of Timeless compromises fitness, we show that it is possible to recover null clones, at least in the context of a *TP53* mutation. (iv) We generate a cell line that is haploinsufficient for Timeless and show that it is exquisitely PARGi sensitive, thus providing a tractable model system to study PARG inhibitor sensitivity. (v) We show that inhibiting PARG stabilizes PAR chains in both resistant and sensitive cells, indicating that the stabilization of nuclear ADPr polymers is not sufficient to cause persistent replication stress. We note that PAR is more abundant in Timeless Δ4 cells, indicating a shift to a hyper-PARylated state. (vi) We show that the Timeless haploinsufficiency also induces sensitivity to drugs targeting the RSR kinases and hydroxyurea, but not to the PARP1/2 inhibitor Olaparib or drugs that directly interact with the DNA. (vii) Inhibiting either PARG or Timeless reduces fork speed, with the combination exacerbating both. Increased EdU foci and PCNA bound to nascent DNA suggests that reduced fork speed is compensated for by increased origin firing (75). Inhibition of PARG also increases fork asymmetry, which is exacerbated by Timeless deficiency. (viii) ADP-ribosylome analysis shows that prolonged PARG inhibition in Timeless-deficient cells results in elevated ADP-ribosylation on a subset of proteins involved in chromatin organization and RNA biogenesis, including Ki67, SAFB and NOP2. (ix) A major finding is that nucleoside supplementation rescues PARGi sensitivity, both in genetically engineered *TIMELESS* cells and in intrinsically sensitive ovarian cancer cells. Importantly, NS reverts all the molecular and cell cycle phenotypes associated with PARGi sensitivity, with the exception of PAR chain accumulation. (x) And finally, we show that inhibition of thymidylate synthase, a key enzyme required for dNTP homeostasis (88), sensitizes several ovarian cancer cells lines to PARGi, as also observed by others (90). In this Discussion, we synthesise these observations to address three questions. Why does inhibition of Timeless confer PARGi sensitivity? Why does NS revert PARGi sensitivity? And, what are the implications for the development of predictive biomarkers to *a priori* identify cancer cells likely to be sensitive to PARG inhibitors?

### Why does inhibition of Timeless confer PARGi sensitivity?

Although *Parg* is essential for mouse embryogenesis (11), in the context of autonomous epithelial cell biology, PARG activity is not essential, illustrated by the fact that non-transformed FNE1 cells proliferate efficiently despite inhibition of PARG activity and accumulation of chromatin-bound PAR chains. Thus, the default state is PARGi resistance. Furthermore, only a subset of cancer cells are PARGi sensitive, presumably due to synthetic lethality with a specific underlying oncogenic vulnerability. The default PARGi-resistant state is dependent on several DNA replication genes including *TIMELESS*, prompting us to ask why does inhibition of Timeless confer PARGi sensitivity, and what does this teach us about the vulnerabilities responsible for intrinsic sensitivity?

Timeless has multiple functions, during both normal and perturbed DNA replication processes, including acting as a fork accelerator, a helicase-polymerase coupler, a fork protector, and a replication checkpoint activator (20–32,75,92–95). Consistent with its fork accelerator function, replisome speed is decreased in haploinsufficienct Timeless Δ4 cells and further exacerbated by PARG inhibition. However, it seems unlikely that global fork slow down alone is responsible for PARGi sensitivity as PARG inhibition also reduces replisome speed in Timeless-proficient cells and, at least in Δ4 cells, this is buffered by increased origin firing (75). One possibility therefore is that another aspect of Timeless dysfunction increases PARG-dependency. For example, if the PARP1-Timeless interaction at the replisome is compromised, then efficient PAR turnover may be essential to ensure PARP1 proximity to the DNA in order to facilitate Okazaki fragment processing (96–98). Or, reduced fork protection may lead to an increase in fork stalling (32), in turn increasing dependency on PARG activity to restart stalled forks by reversing PARP1/2-mediated inhibition of RecQ1 (12,67,68). Note however that, while we did not observe increased fork asymmetry in Δ4 cells in the absence of PARGi, we did observe elevated nuclear PAR in S-phase cells, suggesting persistent activation of PARP1/2. Paradoxically, Timeless Δ4 cells are not sensitive to PARPi suggesting that, if PARGi-sensitivity does reflect a DNA replication problem, PARP1/2 activity is not essential to resolve it. Δ4 cells are however more sensitive to RSR inhibitors, which further supports the existence of a persistent DNA replication problem. That Δ4 cells are not sensitized to all replication stressors, suggests that Timeless’ function as a checkpoint activator is reasonably robust in Δ4 cells.

Another possibility is that rather than PARGi sensitivity being due to global fork slow down, the synthetic Timeless-PARG effect arises due to replication problems at specific hard-to-replicate loci, e.g. G-rich sequences at telomeres, where Timeless facilitates recruitment of helicase activity to unwind G-quadruplex structures (99–102). Interestingly, PARG activity may be required to maintain telomere length, although this appears to be via homology-directed repair mechanisms that support the alternative lengthening of telomeres (ALT) mechanism used by ∼5–15% of cancer cells (103,104). In terms of Timeless’ function as a helicase-polymerase coupler, while a physical coupling is hard to reconcile with a fixed location in front of the DNA helicase (18,19), it possibly reflects the ability of the FPC to reduce helicase speed in response to accumulation of ssDNA behind the replisome (98). Moreover, Timeless dissociates from the chromatin in response to ROS-mediated fork slowing (93), which may also allow it to interact with and accelerate the polymerase. Thus, Timeless deficiency could compromise coupler function, either by failing to slow the helicase and/or accelerate the polymerase, leading to the accumulation of ssDNA, in turn increasing dependency on PARG activity to slow replisome speed and/or restart stalled forks.

Approaching the question from a different perspective, why is the default PARGi-resistant state dependent on Timeless function? In this context, a key observation is the inability of the Timeless SH7A ADP-ribosylation mutant to restore PARGi resistance. While we cannot rule out that one or more of the substituted serines is a phosphorylation site (105), there is compelling evidence that they are modified by ADP-ribose (ADPr) (32,35,57). This observation is provocative when combined with the notion that opposing PARP and PARG activities regulate replisome speed. While speculative, one possibility is that PARG inhibition slows down the replisome via regulating the FPC, possibly by stabilizing PARylation of Timeless itself, which could suppress its accelerator function or promote dissociation from the replisome. PARylation of Timeless has recently been shown to drive Timeless dissociation and degradation by the proteosome, which was suggested to promote stalled fork recovery (32). If Timeless cannot be PARylated due to mutation of ADPr acceptor sites, perhaps replisomes accelerate, thus phenocopying PARPi exposure. And yet, PARPi-induced fork acceleration is not toxic. Furthermore, a Timeless mutant that cannot bind PARP1, and thus cannot be PARylated, exhibited restrained fork progression (32). Perhaps therefore, fork slowing becomes critical in a PARG-deficient environment due to stabilization of PAR chains elsewhere on the chromatin that may interfere with DNA replication. Thus, perhaps PARGi-resistant cells retain the ability to control fork speed and recover stalled forks despite the stabilisation of PAR chains on Timeless or chromatin. By contrast, perhaps PARGi-sensitive cells are less able to regulate replisome speed and/or helicase-polymerase coupling and thus cannot tolerate the presence of PARylated chromatin. If this is the case, then perhaps what studying Timeless teaches us about intrinsic sensitivity is that the underlying vulnerability responsible for the synthetic lethality effect is an inability to slow fork speed and/or retain coupling in response to PARylated chromatin. To test this concept further, important next steps will be studying fork speeds in a broad panel of sensitive and resistant models and identifying the specific ADPr acceptor sites responsible for the SH7A phenotype to facilitate more detailed cellular and biochemical experiments.

### Why does nucleoside supplementation revert PARGi sensitivity?

Pharmacological interventions and genetic alterations that alter nucleotide availability are a major source of replication stress. For example, hydroxyurea or inhibition of ribonucleotide reductase components depletes dNTP pools and potently induces RS (106,107). Driving S-phase entry downstream of MYC, i.e. without concomitant upregulation of nucleotide biogenesis pathways induces RS-driven genomic instability (108). In many cases, NS can revert RS induced by depletion of nucleotide pools. For example, NS restores proliferation of ATR-deficient MEFs (83), and can suppress RS in human pluripotent stem cells (82). Here, we show that NS rescues PARGi sensitivity in both Timeless Δ4 cells and intrinsically sensitive ovarian cancer cells. One possibility is that PARGi sensitivity arises in cells predisposed to RS due to reduced nucleotide pools. And indeed, we show that inhibiting thymidylate synthase can induce PARGi sensitivity. Then in turn, elevating nucleotide availability via NS could explain why RS is alleviated and the dependency on PARG activity diminished. While this may account for intrinsic PARGi sensitivity, it is less obvious how it could account for sensitivity of Δ4 cells. One possibility is that Timeless-deficient cells have diminished ability to couple helicase and polymerase activities, leading to slower replication. While this may be buffered by increased origin firing, it may accelerate nucleotide consumption. If exacerbated by further origin firing to compensate for PARGi-induced fork re-start failure, perhaps nucleotide demand outstrips supply leading to replication catastrophe that can be rescued by NS. Another possibility is that Timeless is involved in sensing nucleotide availability; indeed, one study suggests that Timeless modulates replisome speed in response to fluctuations in dNTP biogenesis via redox signalling and PRDX2 (93). In addition, Tof1-mutant replisomes in yeast progress faster in the presence of hydroxyurea, compared with normal replisomes, consistent with Tof1 (yeast Timeless) regulating replisome speed in response to alterations in nucleotide availability (109). That Δ4 cells are sensitized to hydroxyurea could support a model whereby Timeless senses dNTP availability and/or is required to stimulate dNTP production, for example, via ATR signalling (25,29,42), which has been shown to stimulate S-phase production of dNTPs (42). Note also that resistant ovarian cancer cells can be sensitized to PARGi by co-exposure to hydroxyurea (12).

An alternative possibility is that NS restores PARGi resistance by polymerase acceleration (110). As outlined above, a potential cause of PARGi sensitivity common to Timeless Δ4 cells and intrinsically sensitive models could be the inability to maintain helicase-polymerase coupling in response to PARylated chromatin. If this is the case, promoting coupling by accelerating the polymerase behind the helicase may limit the production of ssDNA, reduce the chance of fork stalling, and thus alleviate the replication stress vulnerability that increases PARG dependency. Distinguishing between these various possibilities will not be easy.

### What are the implications for the development of predictive biomarkers?

Despite the advances described here and elsewhere (8–14,16), our understanding of the molecular mechanisms responsible for intrinsic PARGi sensitivity are not sufficiently advanced to design a robust predictive biomarker for clinical practice. Indeed, multiple mechanisms could account for PARGi sensitivity, including an inability to control replisome speed and/or maintain helicase-polymerase coupling, possibly in response to altered nucleotide availability. Because many upstream pathways could feed into these mechanisms, identifying a single ‘smoking gun’ mutation or gene expression signature that predicts PARGi sensitivity will be challenging. Amplification of *MYC* and *CCNE1*, both common driver events in HGSOC (5), can induce replication stress, so a better understanding of how these events influence replisome dynamics and dNTP bioavailability in the context of PARGi will be important, which in turn will require robust model systems that reflect both HGSOC evolution and PARGi-sensitivity.

## Supplementary Material

zcae030_Supplemental_Files

## Data Availability

Mass spectrometry proteomics data are deposited to the ProteomeXchange Consortium via the Proteomics IDEntifications (PRIDE) (111) partner repository with the dataset identifier PXD049682. iTRAQ data are available in the Supplementary Data.
