# Supplementary material for: Intrinsic PARG inhibitor sensitivity is mimicked by TIMELESS haploinsufficiency and rescued by nucleoside supplementation
Source: NAR Cancer. 2024 Jul 16;6(3):zcae030. doi: 10.1093/narcan/zcae030 (PMC11249981; doi:10.1093/narcan/zcae030)
Supplement: zcae030_Supplemental_Files [file zcae030_supplemental_files.zip › Coulson_Gilmer_Suppl Figures.pdf]

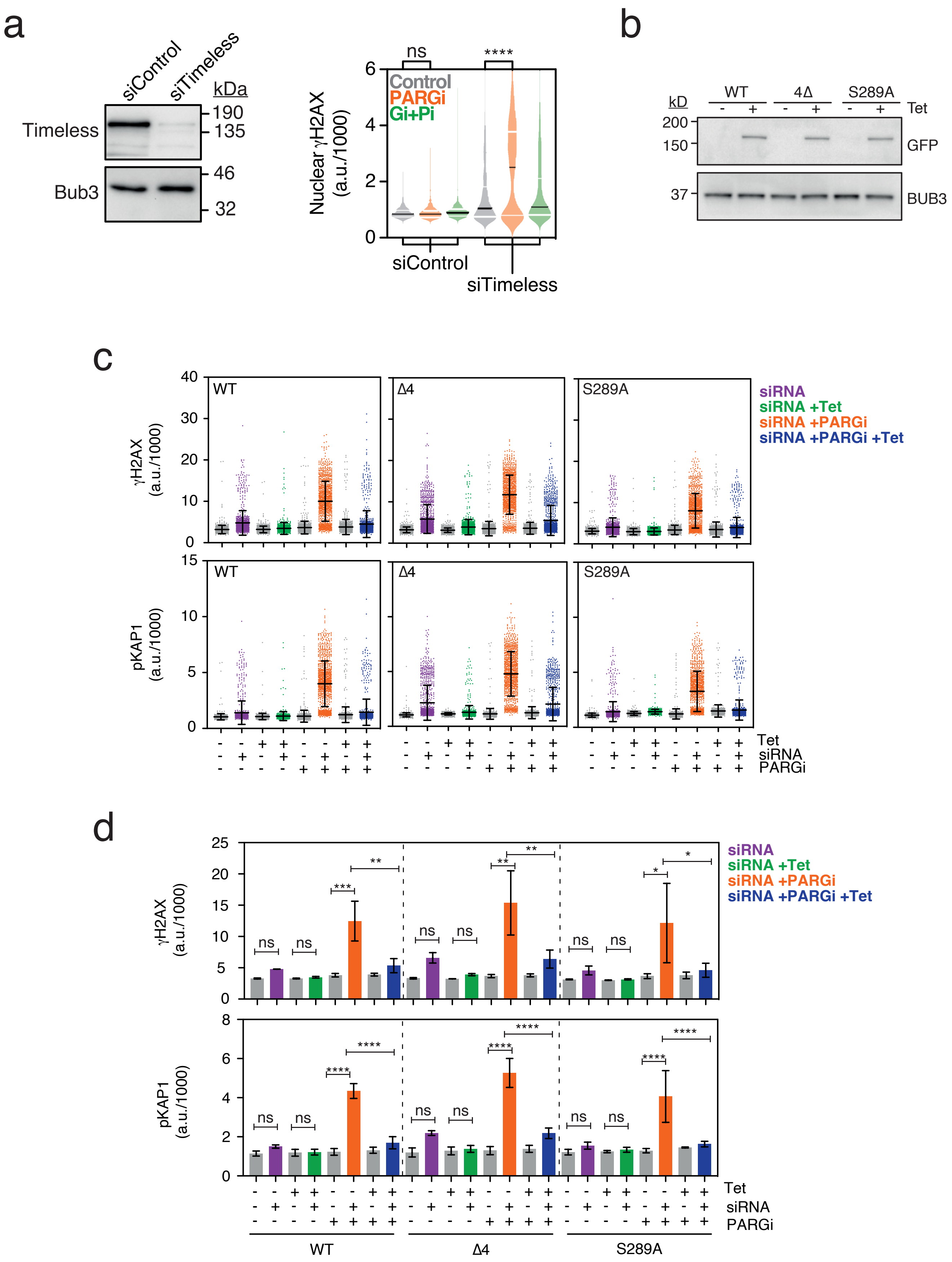
a
siControl
siTimeless
kDa
Timeless
190
135
Bub3
46
32
ns
****
Control
PARGi
Gi+Pi
Nuclear γH2AX (a.u./1000)
siControl
siTimeless
b
kD
WT
4Δ
S289A
Tet
200
150
37
GFP
BUB3
c
WT
Δ4
S289A
γH2AX (a.u./1000)
pKAP1 (a.u./1000)
siRNA
siRNA +Tet
siRNA +PARGi
siRNA +PARGi +Tet
Tet
siRNA
PARGi
d
γH2AX (a.u./1000)
pKAP1 (a.u./1000)
siRNA
siRNA +Tet
siRNA +PARGi
siRNA +PARGi +Tet
Tet
siRNA
PARGi
WT
Δ4
S289A

Figure S1

Figure S1. Probing Timeless function in RKO cells via an RNAi-complementation assay

a) RKO cells are PARGi-resistant in a Timeless dependent manner. Immunoblot shows Timeless levels in parental RKO cells following siRNA transfections. Bub3 is used as a loading control. Violin plot shows quantitation of γH2AX signal in parental RKO cells following siRNA transfections and exposure to 1 µM PARGi or combination of 1 µM PARGi (Gi) and 1 µM PARPi (Pi) for 72 h. Values are from one representative experiment, one-way ANOVA was performed across two independent experiments, ****p<0.0001, ns: p>0.05.

b) Immunoblot of RKO cells transfected with tet-inducible, siRNA-resistant, GFP-tagged Timeless transgenes encoding either WT, Δ4 or S289A proteins. Cells were treated with 500 ng/mL tetracycline for 48h as indicated. Bub3 is used as a loading control.

c) Column scatter plots quantitating pan-nuclear pKAP1 and γH2AX signal in PARGi-treated cells following RNAi complementation. Each symbol represents a single cell, analysing 1000 cells per condition. Bars represent the mean ± SD. Data shown is of one representative experiment.

d) Bar graphs quantitating pan-nuclear pKAP1 and γH2AX signal. Values represent the mean ± SD from two biological replicates. One-way ANOVA with Šídák's multiple comparisons test, *p<0.03, ***p<0.0002, ****p<0.0001, ns: p>0.03.

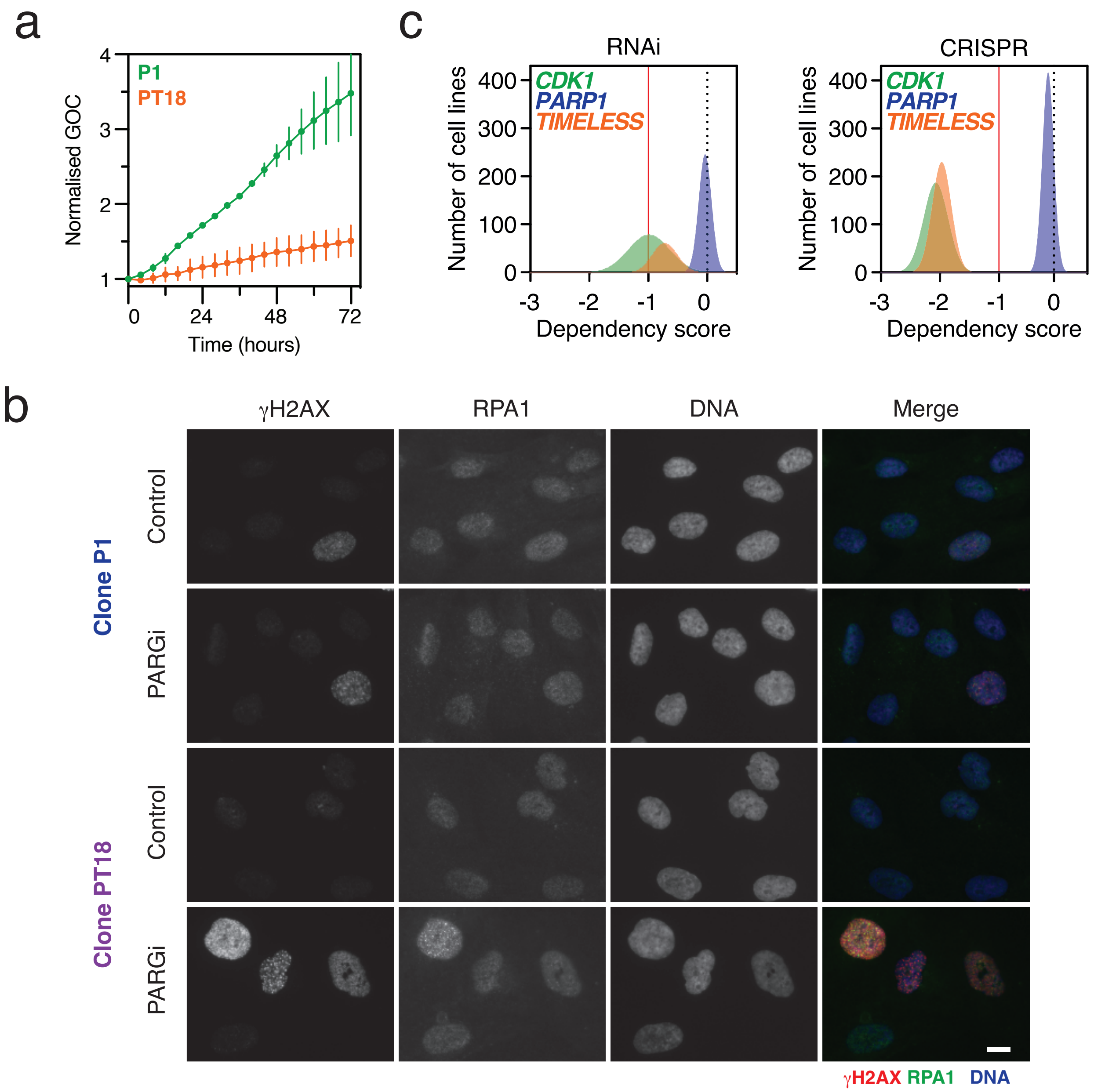
a
P1
PT18
Normalised GOC
Time (hours)
0
24
48
72
1
2
3
4
c
RNAi
CRISPR
CDK1
PARP1
TIMELESS
Number of cell lines
Dependency score
b
γH2AX
RPA1
DNA
Merge
Clone P1
Clone PT18
Control
PARGi
γH2AX RPA1 DNA

Figure S2

Figure S2. Inactivation of *TIMELESS* in p53-deficient FNE1 cells compromises proliferation

a) Line graph measuring normalised green object count (GOC) derived from timelapse microscopy imaging for 72 h, showing proliferation of untreated p53-deficient FNE1 cells (P1, green) and the *TP53*/*TIMELESS* double-mutant (PT18, orange). Values represent the mean ± SD derived from three biological replicates.

b) Immunofluoresence images of p53-deficient FNE1 cells (P1) and the *TP53*/*TIMELESS* double-mutant (PT18) exposed to PARGi and stained to detect γH2AX (red), RPA1 (green) and the DNA (blue). Scale bar 10 µm.

c) Histograms showing RNAi and CRISPR/Cas9 dependency scores for *TIMELESS* in cancer cell lines (dep-map.org). Lower scores indicate increased dependency of a given cell line on the gene of interest, with a score of zero implying not essential. Essential *CDK1* and non-essential *PARP1* shown for reference.

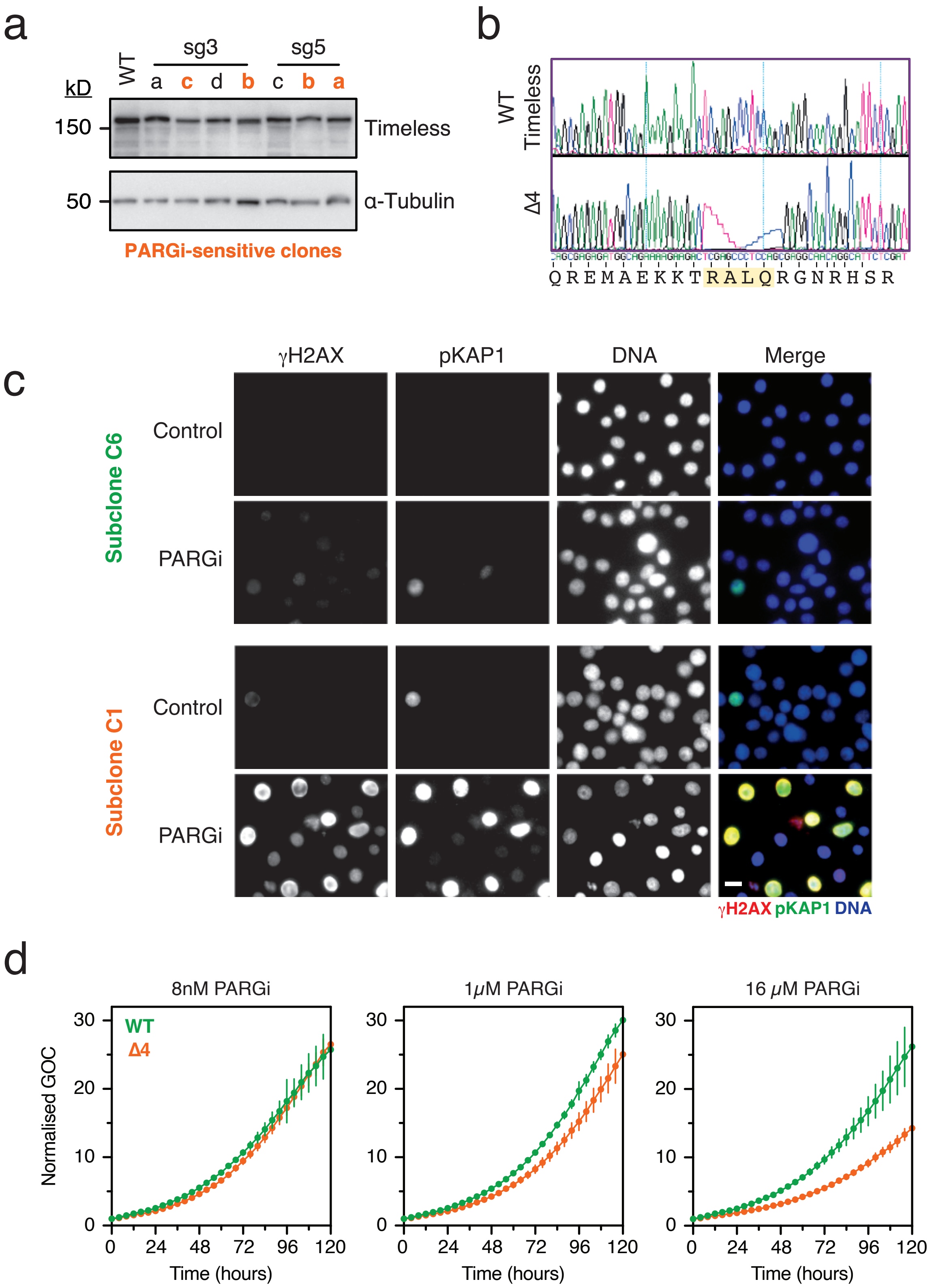
a
kD
150
50
WT
sg3
a c d b
sg5
c b a
Timeless
α-Tubulin
PARGi-sensitive clones
b
WT Timeless
Δ4
Q R E M A E K K T R A L Q R G N R H S R
c
γH2AX
pKAP1
DNA
Merge
Subclone C6
Control
PARGi
Subclone C1
Control
PARGi
γH2AX pKAP1 DNA
d
8nM PARGi
1μM PARGi
16 μM PARGi
WT
Δ4
Normalised GOC
Time (hours)
0 24 48 72 96 120
0 10 20 30

Figure S3

Figure S3. Timeless Δ4 RKO cells proliferate with near-normal kinetics

a) Immunoblot of Timeless expression in CRISPR/Cas9-generated clones using sgRNAs 3 and 5. Tubulin is used as a loading control.

b) Exemplar Sanger sequencing of cloned *TIMELESS* RT-PCR products from parental RKO (WT) and Δ4 clone generated using sgRNA 5, showing a 12 nucleotide in-frame deletion generation of a four amino acid deletion, ΔRALQ.

c) Immunofluoresence images of WT (subclone C6) and Δ4 (subclone C1) RKO cells exposed to PARGi and stained to detect γH2AX (red), pKAP1 (green) and the DNA (blue). Scale bar 10 µm.

d) Line graphs measuring normalised green object count (GOC) derived from timelapse microscopy imaging for 120 h, showing proliferation of WT (green) and Δ4 cells (orange) in the continued presence of 8 nM, 1 µM or 16 µM PARGi. Values show mean ± SE from three biological replicates.

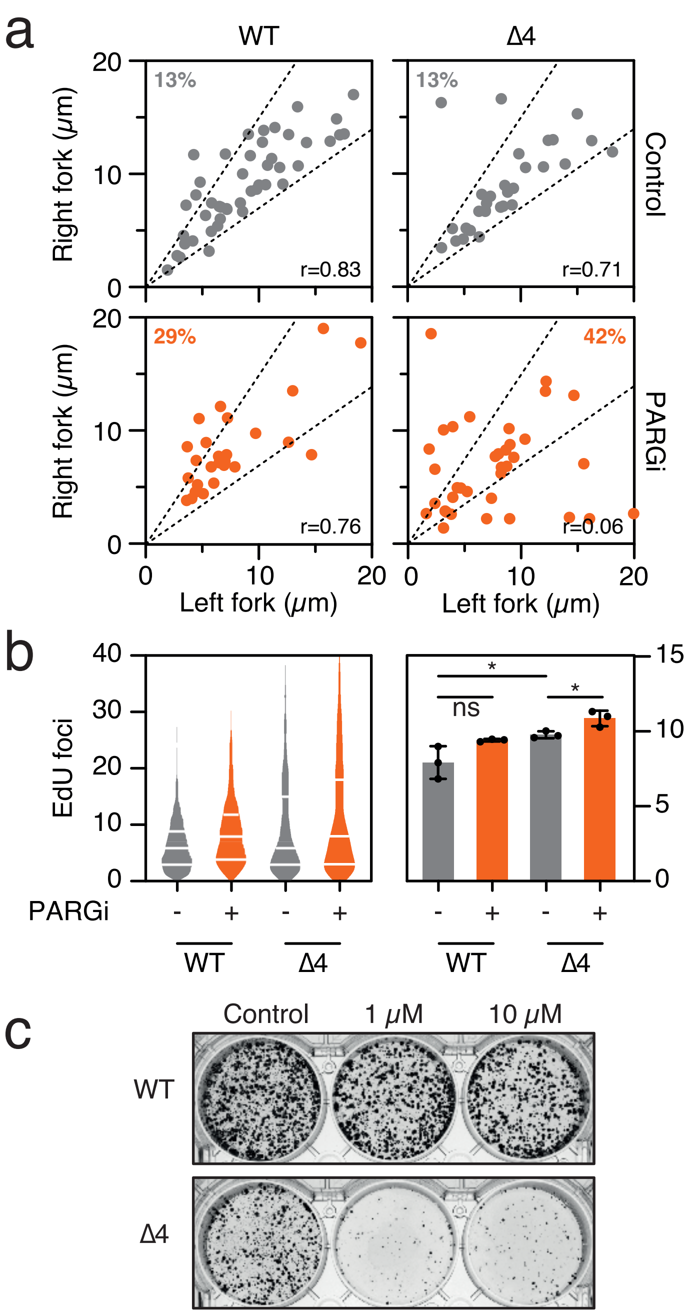
a
WT
Δ4
Right fork (μm)
Left fork (μm)
Control
PARGi
13%
13%
29%
42%
r=0.83
r=0.71
r=0.76
r=0.06
0
10
20
b
EdU foci
PARGi
- + - +
WT
Δ4
ns
*
*
0
5
10
15
40
30
c
Control
1 μM
10 μM
WT
Δ4

Figure S4

Figure S4. PARGi exacerbates fork asymmetry in Timeless Δ4 RKO cells

a) XY scatterplots of cognate left and right DNA fiber lengths in parental WT and Δ4 cells treated with 1 µM PARGi for 24 h. Percentage of asymmetric forks and Spearman correlation noted (origins ≥ 28). Dashed lines indicate asymmetry cut-off defined as >33% difference between sister forks.

b) Graphs quantitating EdU foci in WT or Δ4 cells exposed to 1 µM PARGi for 24 h followed by a 5-minute EdU pulse. Violin plot shows one replicate with median and interquartile ranges. Bar graph shows mean ± SD from three independent replicates, each with minimum of 1000 cells. Two-way ANOVA, *p<0.05, ns: p>0.05.

c) Colony formation assay of parental WT and Δ4 cells treated continuously with 1 µM or 10 µM PARGi.

a

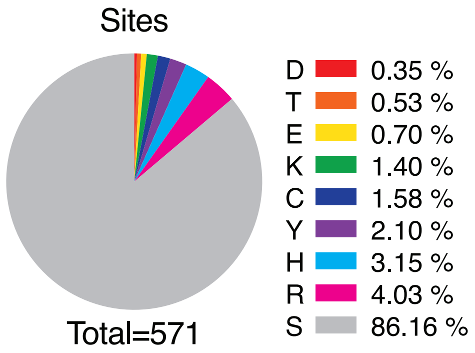

b

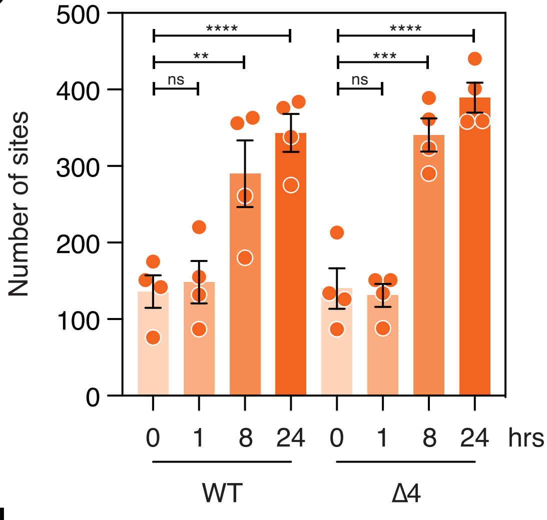

c

	Down	Up	Tested but not significant
Δ4vsWT*	2	21	548
Δ4vsWT.24h**	9	43	519
Δ4vsWT.8h	2	22	547
Δ4vsWT.1h	0	0	571
Δ4vsWT.0h	0	0	571
Δ4.24hvs0h	25	255	291
Δ4.8hvs0h	4	159	408
Δ4.1hvs0h	0	0	571
WT.24hvs0h	9	201	361
WT.8hvs0h	3	115	453
WT.1hvs0h	0	0	571
Both.24hvs0h	25	314	232
Both.8hvs0h	9	229	333
Both.1hvs0h	0	0	571
Interaction***	2	18	551

d

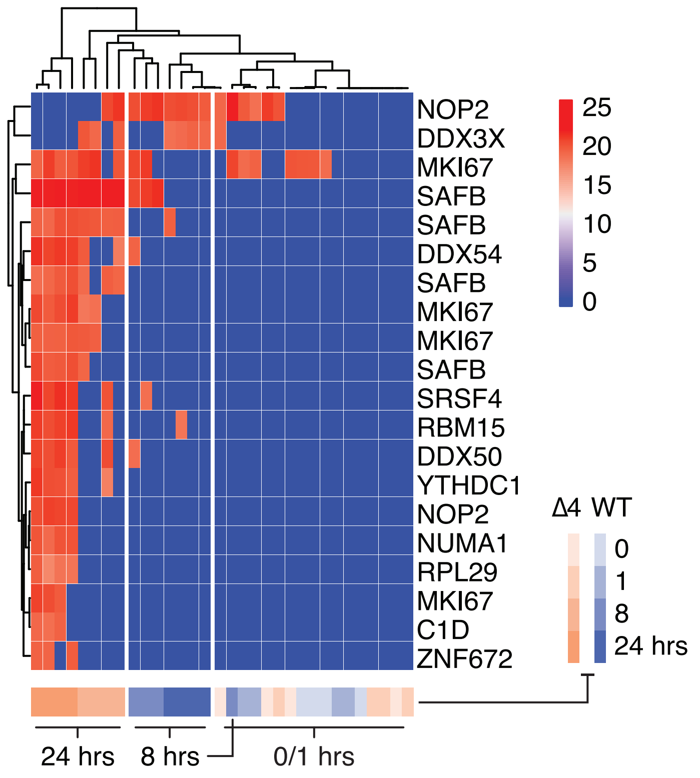

e

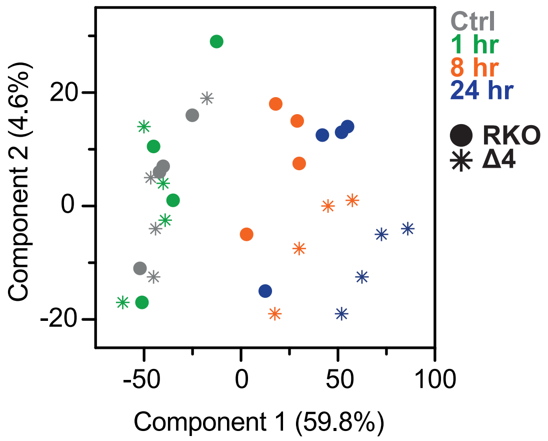

Figure S5

Figure S5. ADP-ribosylome analysis

a) Pie chart showing amino acid distribution of all ADP-ribosylation sites identified across all conditions.

b) Bar graph showing total number of ADP-ribosylation sites identified in parental RKO (WT) and Timeless Δ4 cells treated with 1 µM PARGi for times indicated. Values represent mean ± SD of four independent replicates.

c) Table showing number of acceptor sites with differential ADP-ribosylation intensity for each pairwise comparison. Single asterisk (*) indicates comparison of all Timeless Δ4 timepoints versus all WT timepoints. Double asterisk (**) indicates the comparison used to select acceptor sites included in Figure 5f; Triple asterisk (***) indicates sites identified by the interaction term as sites differentially ADP-ribosylated between Timeless Δ4 and parental cells as a function of PARGi exposure time (i.e. sites with differential intensity between 0 hr and 24 hr for each line, that differ between the lines).

d) Heat map showing unsupervised hierarchical clustering of 20 differentially ADP-ribosylated sites, across 13 unique proteins, identified by the interaction term shown in C. The sample group is indicated by annotation bars below the heatmap, where green and orange indicate WT and Timeless Δ4 respectively. Purple shades from light to dark indicate increasing exposure time to PARGi. Heatmap colours indicate the mass spectrometry intensity of each PARylation site, where red indicates high, and blue indicates low intensity or absence of PARylation.

e) XY scatter plot showing principal component analysis of ADP-ribosylation of WT and Timeless Δ4 cells following treatment with 1 µM PARGi for indicated time. Circles depict WT, stars depict Δ4.

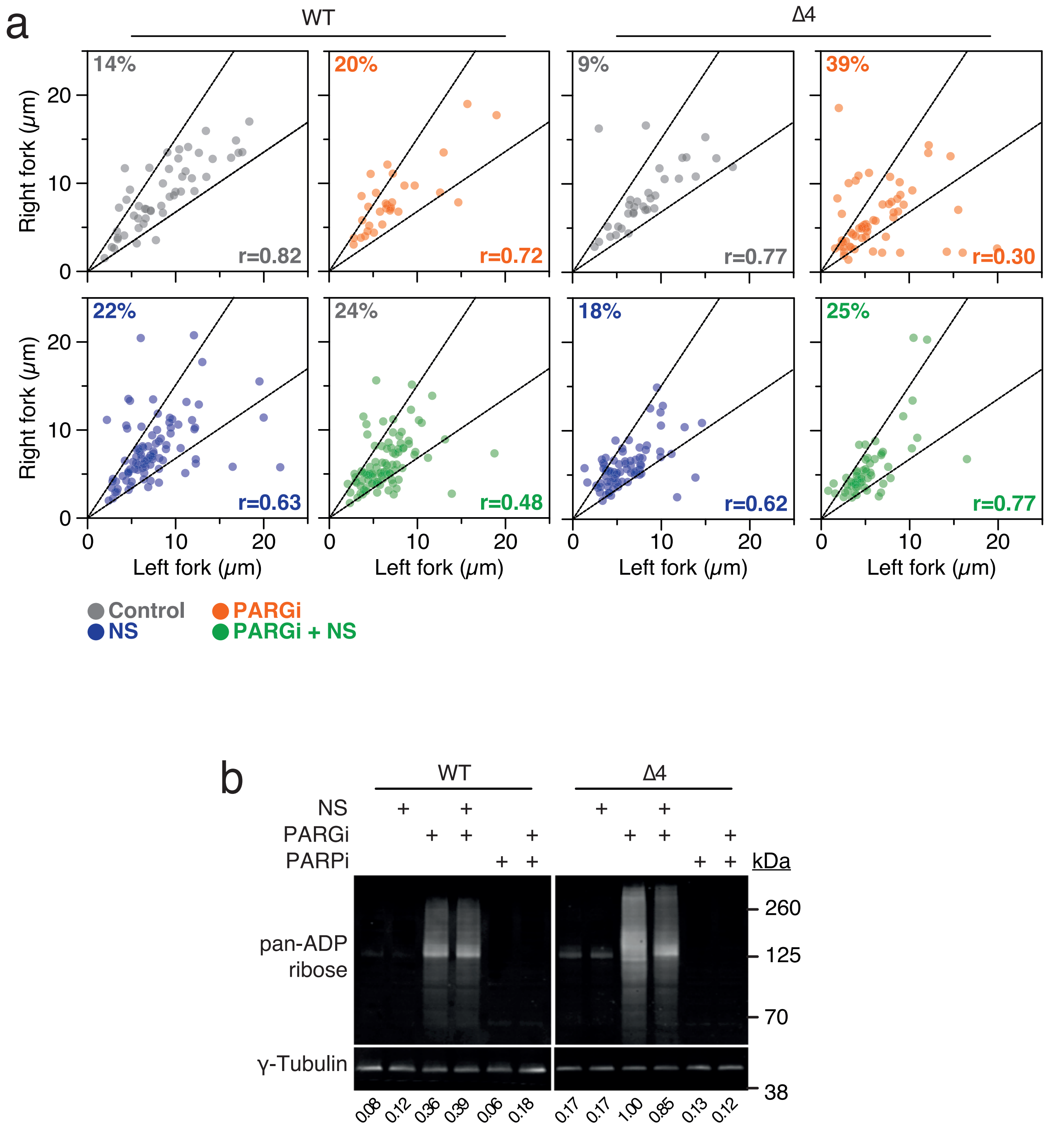
a
WT
Δ4
14%
20%
9%
39%
22%
24%
18%
25%
r=0.82
r=0.72
r=0.77
r=0.30
r=0.63
r=0.48
r=0.62
r=0.77
Right fork (μm)
Left fork (μm)
Control
PARGi
NS
PARGi + NS
b
WT
Δ4
NS
PARGi
PARPi
kDa
260
125
70
38
pan-ADP ribose
γ-Tubulin
0.08 0.12 0.36 0.39 0.06 0.18
0.17 0.17 1.00 0.85 0.13 0.12

Figure S6

Figure S6. Nucleoside supplementation ameliorates PARGi-induced fork asymmetry in *TIMELESS* Δ4 cells

a) XY scatterplots of cognate left and right DNA fiber lengths in parental RKO (WT) and Δ4 cells treated with indicated treatments for 24 h. Percentage of asymmetric forks and Spearman correlation (origins ≥ 34) noted. Dashed lines indicate asymmetry cut-off defined as >33% difference between sister forks.

b) LiCOR-based immunoblotting for pan-ADP ribose (PAR) and γ-Tubulin in WT and Δ4 cells. Cells were treated with the indicated treatments for 72 h. Images are from one experiment. Mean quantitation of three biological replicates given (see Figure 6G).

PARGi concentration is 1 µM, NS concentration is 0.1 mM, control treatment is DMSO.

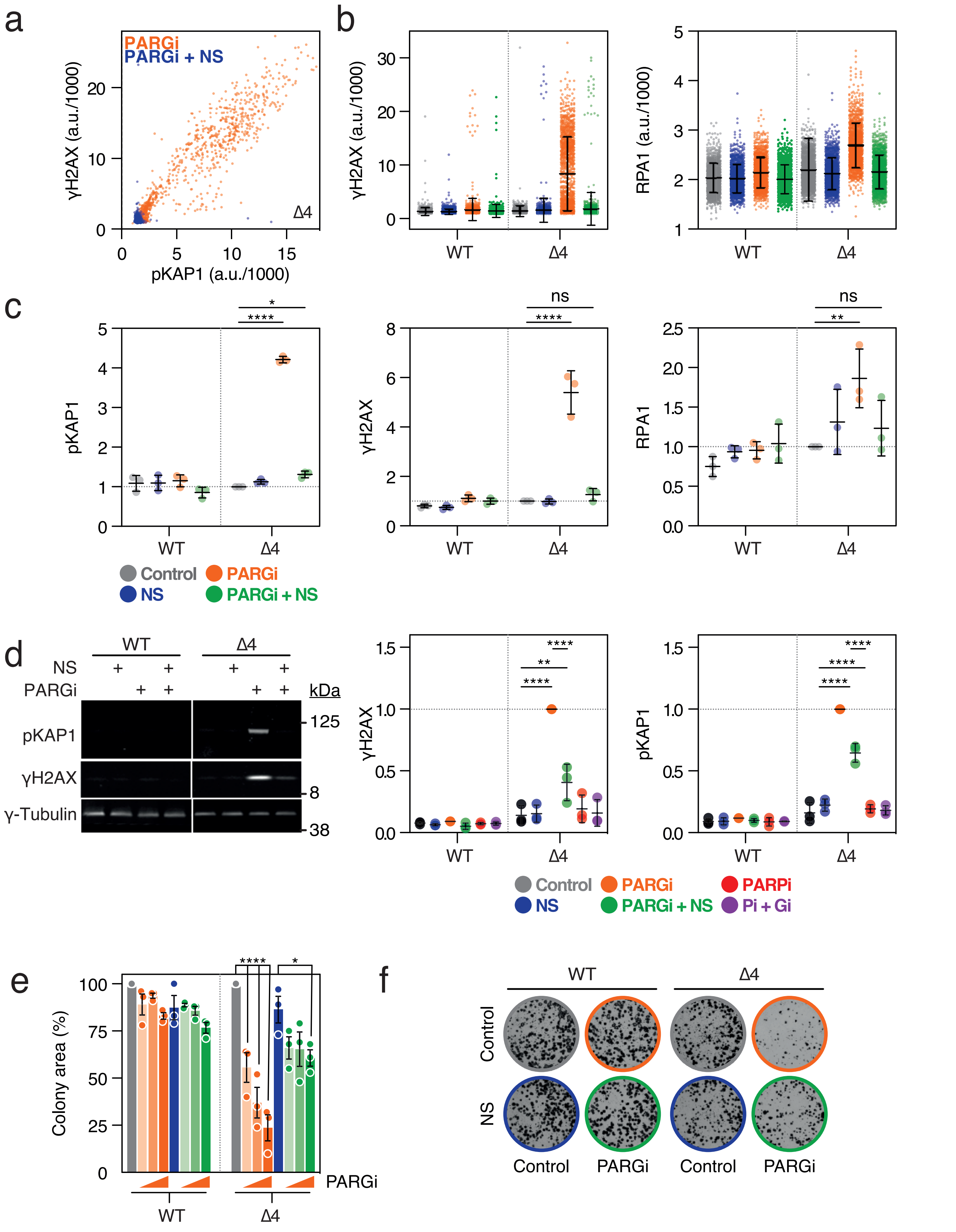
a
PARGi
PARGi + NS
γH2AX (a.u./1000)
pKAP1 (a.u./1000)
Δ4
b
γH2AX (a.u./1000)
RPA1 (a.u./1000)
WT
Δ4
c
pKAP1
γH2AX
RPA1
ns
*
****
**
Control
PARGi
NS
PARGi + NS
d
WT
Δ4
NS
PARGi
kDa
pKAP1
γH2AX
γ-Tubulin
125
8
38
PARPi
Pi + Gi
e
Colony area (%)
PARGi
f
Control
NS
Control
PARGi

Figure S7

Figure S7. Nucleoside supplementation ameliorates PARGi sensitivity in *TIMELESS* Δ4 cells

a) XY dot plot γH2AX/pKAP1 in Δ4 cells treated as indicated for 72 h. Data derived from 1000 cells. See also Figure 6b and S7b.

b) Column scatter plots quantitating nuclear γH2AX and RPA immunofluorescence intensity in parental RKO (WT) and Δ4 cells, treated for 72 h. Data derived from 1000 cells, lines represent mean ± SD. See panel (c) for colour key.

c) Quantification of nuclear pKAP1, γH2AX and RPA immunofluorescence intensity in parental WT and Δ4 cells treated as indicated for 72 h. Lines represent mean and SE of three biological replicates. Two-way ANOVA with Tukey's post-hoc, where ****$p<0.0001$, **$p<0.01$, *$p<0.05$, ns: $p>0.05$. Data for Δ4 cells are summarised in Figure 6C.

d) LiCOR-based immunoblot analysis of pKAP1, γH2AX in WT and Δ4 cells treated with the indicated agents for 72 h. Image on left shows representative example, with γ-Tubulin is used as a loading control. Scatter column plot on right quantitates values adjusted for total protein stain and normalised to PARGi-treatment of Δ4 cells. Two-way ANOVA with Tukey's post-hoc, where ****$p<0.0001$, **$p<0.01$. Data are the mean of three biological replicates, error bars represent SEM. Data for Δ4 cells are summarised in Figure 6D.

e) Bar graph of colony area as a percentage of untreated control (grey) showing PARGi only (orange), NS only (blue) and PARGi plus NS (green) with PARGi concentrations of 0.5, 1.0 and 2.0 µM. Values show mean ±SEM of three biological replicates. Significant comparisons versus control or NS alone are indicated (Two-way ANOVA, Tukey's post-hoc test), ****$p<0.0001$, *$p<0.05$.

f) Exemplar colony formation assay for parental WT and Δ4 cells treated continuously with PARGi ± NS. See Figure 6F for quantification of three biological replicates.

Unless otherwise indicated PARGi concentration is 1 µM, NS concentration is 0.1 mM, PARPi concentration is 1 µM, Control treatment is DMSO.

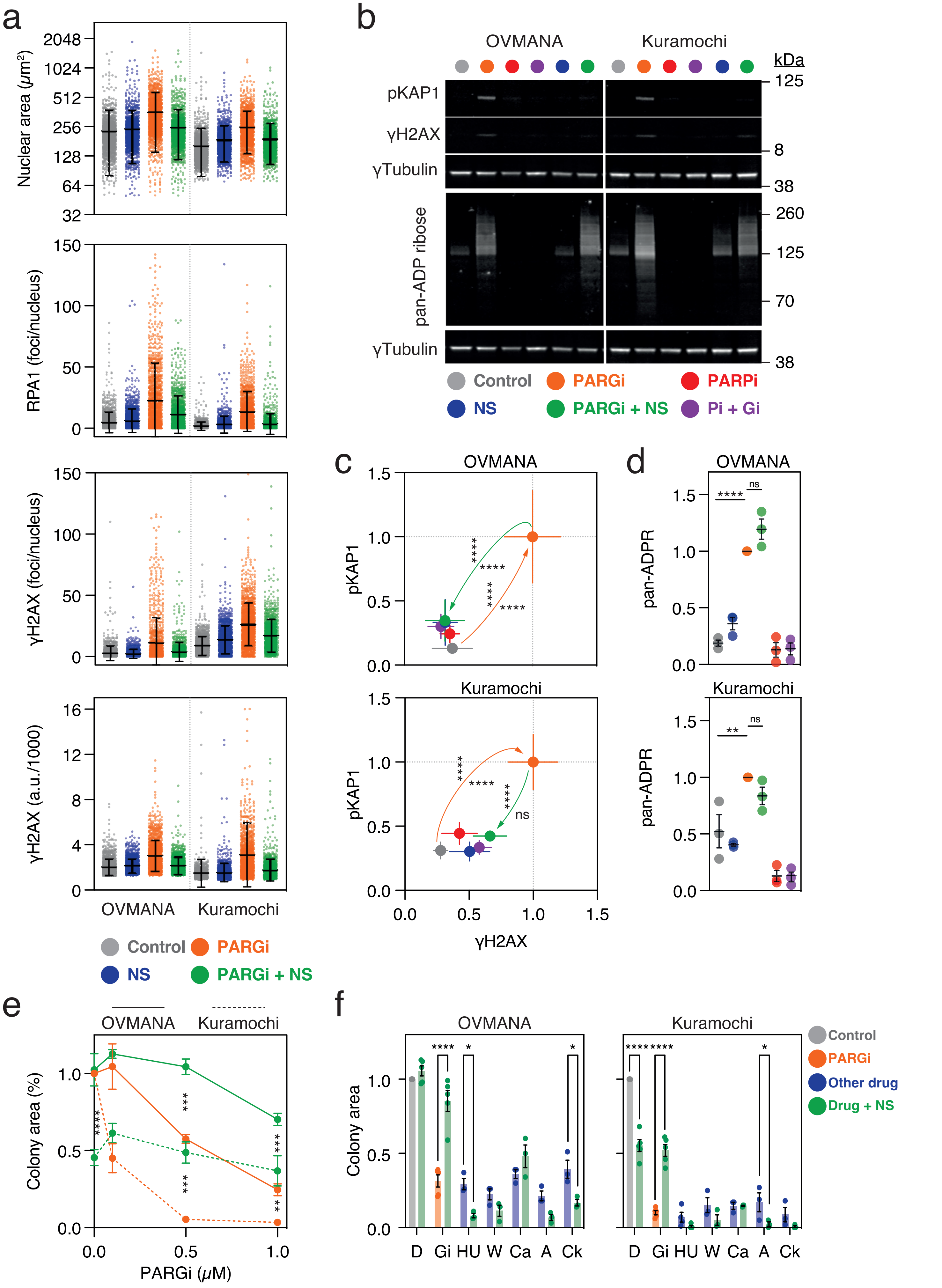
a
Nuclear area (μm²)
2048
1024
512
256
128
64
32
RPA1 (foci/nucleus)
150
100
50
0
γH2AX (foci/nucleus)
γH2AX (a.u./1000)
16
12
8
4
OVMANA
Kuramochi
Control
PARGi
NS
PARGi + NS
b
OVMANA
Kuramochi
kDa
pKAP1
γH2AX
γTubulin
pan-ADP ribose
125
8
38
260
70
Control
PARGi
PARPi
NS
PARGi + NS
Pi + Gi
c
pKAP1
γH2AX
1.5
1.0
0.5
0.0
****
ns
d
pan-ADPR
**
e
Colony area (%)
PARGi (μM)
***
f
Colony area
D
Gi
HU
W
Ca
A
Ck
*
Other drug
Drug + NS

Figure S8

Figure S8. Nucleoside supplementation rescues intrinsic PARGi sensitivity

a) Column scatter plots quantitating nuclear area, RPA1 foci, γH2AX foci and intensity in OVMANA and Kuramochi cells. ≥762 cells plotted per condition, from one experiment. Mean and SD indicated.

b) LiCOR-based immunoblot of pKAP1, γH2AX, and pan-ADP ribose in OVMANA and Kuramochi cells treated as indicated for 72 h. γ-Tubulin is a loading control. Single representative experiment of multiple biological replicates (n≥3).

c) XY plots quantitating of LiCOR immunoblots of pKAP1 and γH2AX, adjusted for total protein stain and normalised to PARGi-treatment. Values show mean ± SEM, n≥3. Two-way ANOVA with Tukey's post-hoc test, selected comparisons shown, ****p<0.0001, ns: p>0.05.

d) Column scatter plots quantitating LiCOR-based immunoblots of pan-ADP ribose, adjusted for total protein stain, and normalised to PARGi-treatment. Values show mean ± SEM n≥3. One-way ANOVA with Šídák's post-hoc test, ****p<0.0001, **p<0.01, ns: p>0.05.

e) Line graphs of percentage colony area for PARGi-treated (orange) and PARGi plus NS-treated cells (green) over a range of PARGi concentrations in OVMANA (solid lines) and Kuramochi (dashed lines). Values show mean ± SEM from n≥3 biological replicates. Significant comparisons shown (Two-way ANOVA, Šídák's post-hoc test), ****p<0.0001, ***p<0.001, **p<0.01.

f) Bar graphs showing percent colony area for OVMANA and Kuramochi, normalised to respective controls, following continuous drug exposures indicated ±0.25 mM NS. Drug concentrations as follows for OVMANA: PARGi 0.5 µM, hydroxyurea (HU) 250 µM, Wee1i 0.3 µM, Carboplatin 0.4 µM, ATRi 0.5 µM, CHK1i 0.16 µM. For Kuramochi: PARGi 0.25 µM, hydroxyurea (HU) 100 µM, Wee1i 0.6 µM, Carboplatin 0.6 µM, ATRi 0.25 µM, CHK1i 0.2 µM. Values represent mean ± SEM of ≥3 biological replicates. Significant comparisons shown (One-way ANOVA, Šídák's post-hoc test), **** p<0.0001, * p<0.05. n≥3.

Unless otherwise indicated PARGi concentration is 1 µM, NS concentration is 0.25 mM, PARPi concentration is 1 µM, Control treatment is DMSO.
